# Alzheimer‐like tau accumulation in dentate gyrus mossy cells induces spatial cognitive deficits by disrupting multiple memory‐related signaling and inhibiting local neural circuit

**DOI:** 10.1111/acel.13600

**Published:** 2022-03-31

**Authors:** Shihong Li, Qiuzhi Zhou, Enjie Liu, Huiyun Du, Nana Yu, Haitao Yu, Weijin Wang, Mengzhu Li, Ying Weng, Yang Gao, Guilin Pi, Xin Wang, Dan Ke, Jian‐Zhi Wang

**Affiliations:** ^1^ Department of Pathophysiology School of Basic Medicine Key Laboratory of Education Ministry of China/Hubei Province for Neurological Disorders Tongji Medical College Huazhong University of Science and Technology Wuhan China; ^2^ Department of Pathology The First Affiliated Hospital of Zhengzhou University Zhengzhou China; ^3^ Department of Physiology School of Basic Medicine and Tongji Medical College Huazhong University of Science and Technology Wuhan China; ^4^ Co‐Innovation Center of Neuroregeneration Nantong University Nantong China

**Keywords:** Alzheimer's disease, hippocampus, hTau N368, mossy cell, spatial memory

## Abstract

Abnormal tau accumulation and spatial memory loss constitute characteristic pathology and symptoms of Alzheimer disease (AD). Yet, the intrinsic connections and the mechanism between them are not fully understood. In the current study, we observed a prominent accumulation of the AD‐like hyperphosphorylated and truncated tau (hTau N368) proteins in hippocampal dentate gyrus (DG) mossy cells of 3xTg‐AD mice. Further investigation demonstrated that the ventral DG (vDG) mossy cell‐specific overexpressing hTau for 3 months induced spatial cognitive deficits, while expressing hTau N368 for only 1 month caused remarkable spatial cognitive impairment with more prominent tau pathologies. By in vivo electrophysiological and optic fiber recording, we observed that the vDG mossy cell‐specific overexpression of hTau N368 disrupted theta oscillations with local neural network inactivation in the dorsal DG subset, suggesting impairment of the ventral to dorsal neural circuit. The mossy cell‐specific transcriptomic data revealed that multiple AD‐associated signaling pathways were disrupted by hTau N368, including reduction of synapse‐associated proteins, inhibition of AKT and activation of glycogen synthase kinase‐3β. Importantly, chemogenetic activating mossy cells efficiently attenuated the hTau N368‐induced spatial cognitive deficits. Together, our findings indicate that the mossy cell pathological tau accumulation could induce the AD‐like spatial memory deficit by inhibiting the local neural network activity, which not only reveals new pathogenesis underlying the mossy cell‐related spatial memory loss but also provides a mouse model of Mossy cell‐specific hTau accumulation for drug development in AD and the related tauopathies.

## Graphical abstract

Mossy cell pathological tau accumulation induces the Alzheimer disease (AD)‐like spatial memory deficit by inhibiting the local neural network activity. The ventral mossy cell‐specific overexpressing hTau disrupted multiple AD‐associated signaling pathways, including reduction of synapse‐associated protein, inhibition of AKT and activation of glycogen synthase kinase (GSK‐3β).

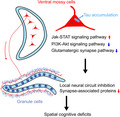

## 1 INTRODUCTION

Alzheimer disease (AD) is the most common neurodegenerative disorder characterized clinically by spatial memory loss in the early stage (Scheltens et al., 2021), but the underlying molecular mechanisms are not fully understood. It is well recognized that the hippocampal tri‐synaptic circuit, that is, entorhinal cortex (EC)‐DG‐CA3‐CA1 linked respectively by perforant fibers, mossy fibers, and Schaffer collateral fibers, plays a crucial role in spatial cognitive functions. The impairments at any node of this tri‐synaptic circuit can cause or at least contribute to the AD‐like spatial cognitive deficits.

By virtue of its unique plasticity and its integrative properties, compelling evidence suggests the enigmatic hilar neurons in the hippocampal dentate gyrus (DG), an intermediate step of the tri‐synaptic circuit, play a crucial role in memory encoding, retrieval and discrimination, and experimental lesions to the hippocampal DG disrupts spatial memory formation (Hainmueller & Bartos, 2020). The DG is composed of several different cell types, including excitatory mossy cells and granule cells, the inhibitory GABAergic interneurons, the glial cells, and different stages progenitor cells. While the number of mossy cells accounts for over 30% of the total hilar cells in rodents, suggesting their importance (Scharfman, 2018). The mossy cells in hilar are largely composed of glutamatergic neurons that innervate both excitatory granular neurons and inhibitory GABAergic neurons within the DG (Scharfman, 2016; Scharfman & Myers, 2012). The mossy cells in hilar have unique structure and physiological properties, and they control DG cell excitability and share a role in pattern separation by interacting with proximal CA3 pyramidal cells (Henze & Buzsaki, 2007; Jinde et al., 2013; Scharfman, 2016; Sloviter et al., 2012). The mossy cells in hilar also control spontaneous convulsive seizures and spatial memory (Bui et al., 2018). Deletion or degeneration of mossy cells can cause a transient dentate granule cell hyperexcitability and impair pattern separation, leading to anxiety and impaired contextual discrimination (Jinde et al., 2012). Recent studies also reveal a pivotal role of mossy cells in regulating DG‐dependent spatial memory (Botterill, Vinod, et al., 2021; Fredes et al., 2021; GoodSmith et al., 2019).

Tau is a neuronal microtubule‐associated protein with the major function in promoting microtubule assembly and stabilizing microtubules (Cleveland et al., 1977; Fellous et al., 1977; Wang & Liu, 2008; Weingarten et al., 1975). In patients with AD and the related tauopathies, the hippocampal formation is largely impacted by accumulation of the hyperphosphorylated tau, accompanied by the reduced synapse number and decreased adult neurogenesis and neurodegeneration (Hamilton et al., 2015). We have recently reported that hTau accumulation in the hilar GABAergic interneurons impairs adult hippocampal neurogenesis (AHN) and spatial memory (Zheng et al., 2020). Here, we report a prominent hTau accumulation in the hilar mossy cells in 3xTg‐AD mice, and the accumulated hTau proteins were largely composed of the truncated hTau N368, a more toxic species of hTau. However, contribution of the mossy cell hTau accumulation, especially hTau N368, to the functional deficiency of the hippocampus and the hippocampus‐dependent cognitive functions is unclear.

In the present study, we first confirmed accumulation of the truncated hTau N368 in the DG mossy cells of 3xTg‐AD mice. Further studies by mossy cell‐specific overexpressing full‐length wild‐type hTau or the truncated hTau N368 demonstrated that both wild‐type hTau and hTau N368 accumulation induced spatial cognitive deficits, while the deficit caused by hTau N368 was much faster than the full‐length hTau. By in vivo electrophysiological and optic fiber recording, we observed that the ventral DG (vDG) mossy cell‐specific overexpressing hTau N368 disrupted theta oscillations with inactivation of local neural network in the dorsal DG subset. Transcriptomic data revealed that hTau N368 disrupted multiple AD‐associated signaling pathways in the hTau N368‐loaded mossy cells, including the remarkably reduced levels of synapse‐associated proteins, inhibition of AKT and activation of glycogen synthase kinase‐3β (GSK‐3β). Finally, chemogenetic activating mossy cells in DG attenuated hTau N368‐induced spatial cognitive deficits.

## 2 RESULTS

### 2.1 Hyperphosphorylated tau is most predominantly detected in DG mossy cells of 3xTg‐AD mice

Previous studies demonstrated that 7‐month‐old 3xTg‐AD mice had spatial cognitive deficits. To explore the involvement of tau, we examined the distribution of hyperphosphorylated tau (pTau) in the hippocampal DG subset of 3xTg‐AD mice. A predominant accumulation of the phosphor‐Thr205 (pT205) and phosphor‐Thr231 (pT231) was detected in the hilus region of 7‐month‐old 3xTg‐AD mice (Figure S1a,c). Interestingly, the majority of these pT205‐ and pT231‐positive cells was co‐labelled with calretinin (Fujise et al., 1998; Liu et al., 1996), a marker of mossy cell (Figure S1b,d). Thereafter, we focused on the role of mossy cell‐specific pTau accumulation in the AD‐like spatial cognitive impairment and the underlying mechanisms.

### 2.2 Mossy cell‐specific overexpressing wild‐type full‐length hTau induces cognitive deficits with truncated hTau N368 accumulation‐dependent manner

**FIGURE 1:**
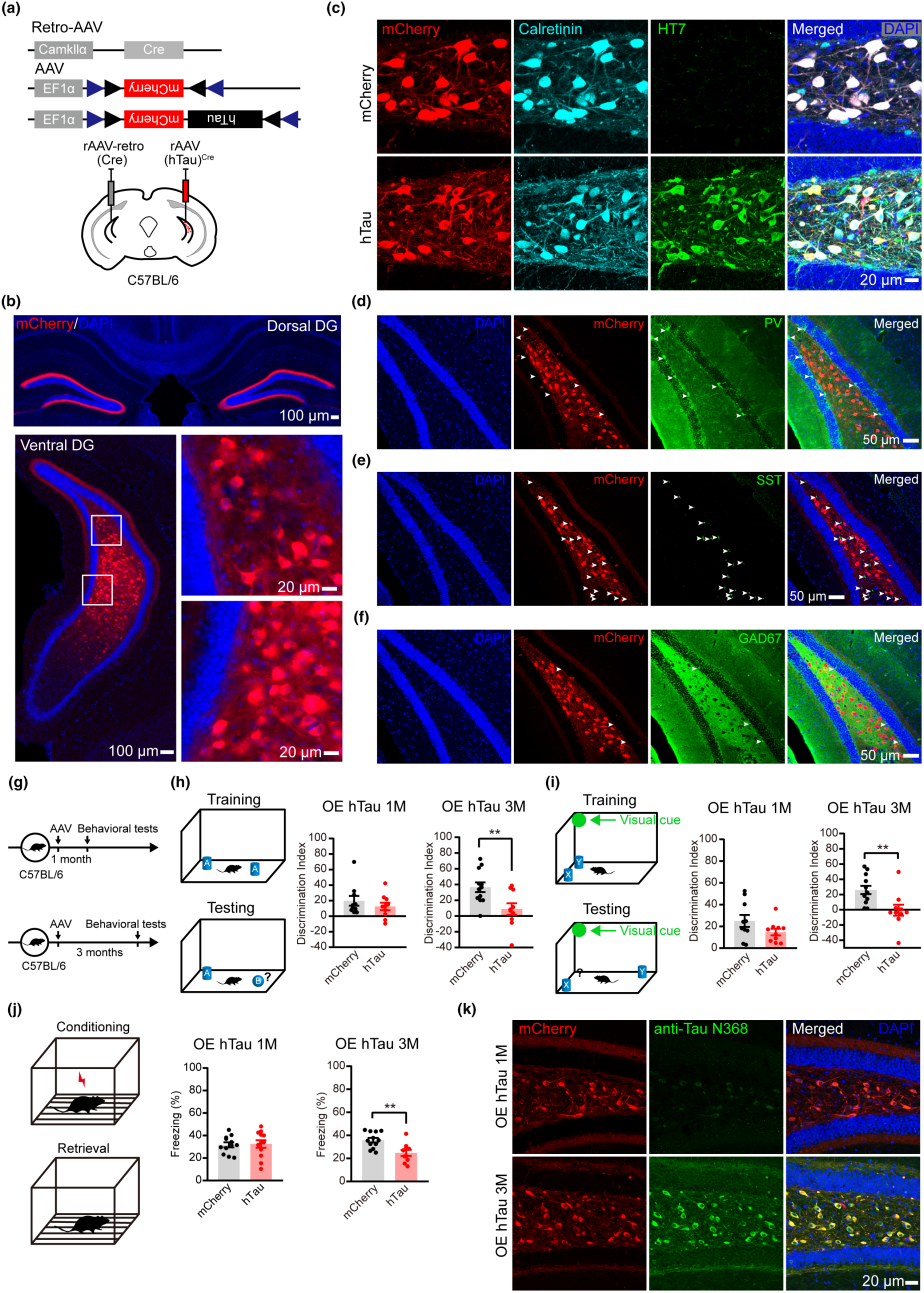
Mossy cell‐specific overexpressing hTau induces cognitive deficits with truncated hTau N368 accumulation‐dependent manner. (a) Strategies used for mossy cell‐specific overexpressing hTau in C57BL/6 mice: recombinant adeno‐associated virus (rAAV)/retro‐Cre was stereotaxically infused into one side and rAAV‐hTau^Cre^ was simultaneously infused at the opposite side of the ventral hippocampal dentate gyrus (DG) subsets. (b–f) Representative images shown mCherry expression pattern (b) and confirmed mossy cell‐specific overexpression of hTau in ventral DG measured by co‐labeling of HT7 (specifically reacts with hTau) and calretinin (mossy cell marker) (c). The mCherry was not co‐labeled with PV (arrows in panel d), SST (arrows in panel e), and GAD67 (arrows in panel f). Scale bars were indicated in each image. (g–i) Mossy cell overexpressing hTau for 3 months but not for 1 month in C57BL/6 mice decreased discrimination index. In this paradigm, mice were infused with empty vector (mCherry) or hTau for 1 or 3 months and then were tested to discriminate the novel object (object B) from familiar object (object A) (h), or the object (object Y) moved to a novel place (i). Unpaired *t* tests, **p < 0.01, *n* = 10–12 mice in each group. (j) Mossy cell overexpressing hTau for 3 months but not for 1 month impaired contexture memory shown by the decreased freezing during contextual fear conditioning retrieval tests. In this paradigm, the mice were allowed to explore for 3 min in a conditioning chamber, then exposed to three times foot shocks (0.65 mA, 2 s each, and 1 min rest in between), and were removed from the chamber 10 s later. After 24 h, the mice were put into the same training chamber without shocks, and the total freezing time in 3 min was measured. Unpaired *t* tests, **p < 0.01, *n* = 10–12 mice in each group. (k) Mossy cell overexpressing hTau for 3 months induced accumulation of the truncated pathological hTau N368 detected by anti‐Tau N368. Scale bar, 20 μm. Data were represented as mean ± *SEM*

To explore whether the AD‐like human tau (hTau) accumulation in mossy cells affects cognitive functions, we first used a strategy to achieve a unilateral mossy cell‐specific overexpression of hTau in C57BL/6 mice, as reported in a previous study (Ratzliff et al., 2004). A retrograde variant of recombinant adeno‐associated virus (rAAV2‐retro) expressing Cre‐recombinase (Tervo et al., 2016) was infused into the vDG; simultaneously, a Cre‐dependent rAAV‐expressing vector (AAV‐EF1α‐DIO‐hTau‐mCherry or AAV‐EF1α‐DIO‐mCherry) was infused into the contralateral vDG subset (Figure 1a). After 1 or 3 months, the expression and distribution of mCherry were detected (Figure 1b). As expected, the mCherry was prominently co‐labeled with calretinin (a mossy cell marker in the hilus region of DG), but not with PV, GAD67‐ and SST (Figure 1c–f). These data indicate that the strategy used for mossy cell‐specific overexpression of hTau in C57BL/6 mice is efficient.

Then, we studied the role of mossy cell hTau accumulation in cognitive functions. By object recognition test (ORT), a pronounced deficit was detected only at 3 months but not at 1 month after mossy cell overexpression of hTau (Figure 1g,h). In the hippocampus‐dependent object location test (OLT), the mice with mossy cell‐overexpressing hTau for 3 months failed to discriminate the displaced object from an unmoved object, indicating spatial memory deficit (Figure 1i). The contextual fear conditioning test (CFC) data showed that mossy cell overexpression of hTau for 3 months decreased freezing time during the 3‐min memory test done at 24 h after the training, which confirmed the contextual memory impairment by mossy cell hTau accumulation (Figure 1j). No locomotor disability or anxiety‐like behavior was detected in open field test and the elevated plus maze test (Figure S2). These data suggest that mossy cell‐specific accumulation of full‐length wild‐type hTau in C57BL/6 mice induces spatial memory deficits in a time‐dependent manner.

Different cleaved tau fragments, including hTau N368, have been detected respectively in the brains of AD patients, the AD‐like transgenic mouse models and during aging (Zhang et al., 2014). By immunofluorescence staining, we also detected a time‐dependent accumulation of hTau N368 after mossy cell overexpression of full‐length hTau (Figure 1k), which was correlated with the observed memory impairments (Figure 1h–j). These data together suggest that mossy cell accumulation of the cleaved hTau N368 may be responsible for the observed cognitive deficits in the mice. Thereafter, we focus on the role of mossy cell‐specific accumulation of hTau N368.

### 2.3 Mossy cell‐specific overexpressing hTau N368 for only 1 month induces cognitive deficits with a more prominent AD‐like pathological tau accumulation

**FIGURE 2:**
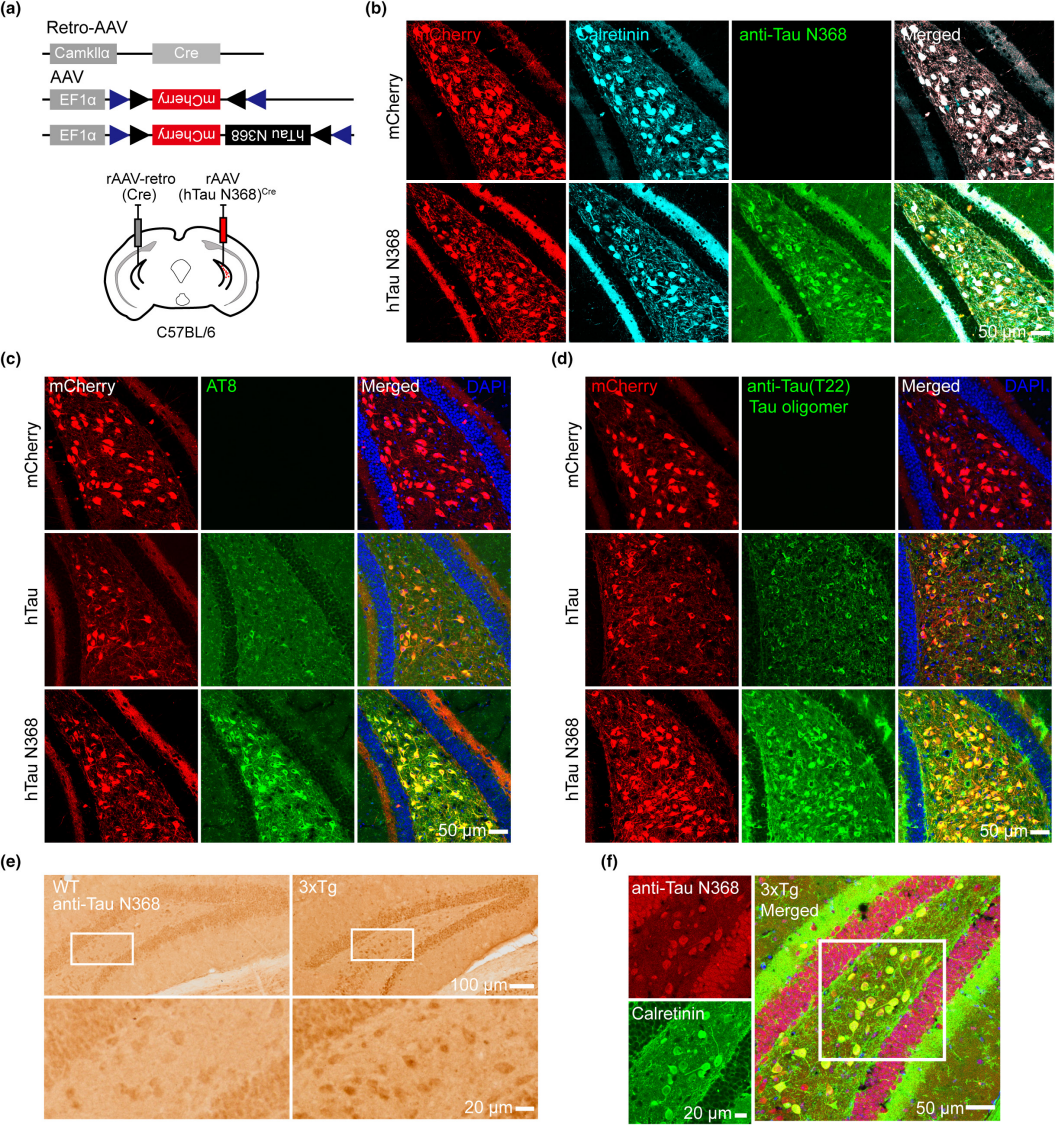
Mossy cell‐specific overexpressing hTau N368 for 1 month causes Alzheimer disease (AD)‐like pathological tau accumulation. (a) Strategies used for mossy cell‐specific overexpressing hTau N368 in C57BL/6 mice. The recombinant adeno‐associated virus (rAAV)/retro‐Cre was stereotaxically infused at one side and rAAV‐hTau N368^Cre^ was simultaneously infused at the contralateral side of the hippocampal dentate gyrus subsets. (b) Representative images confirming mossy cell overexpression of hTau N368 measured by co‐labelling of mCherry, anti‐Tau N368, and calretinin. (c, d) Representative co‐immunofluorescence images showing that mossy cell‐overexpressing hTau N368 induced more prominent accumulation of the AD‐like hyperphosphorylated (AT8) and oligomerized (T22) hTau than overexpressing full‐length hTau. (e, f) Representative images showing prominent accumulation of the AD‐like pathologically truncated Tau N368 in hilus of 10‐month 3xTg‐AD mice (e), and most Tau N368‐positive cells in hilus were identified as mossy cells, as indicated by co‐labeling of anti‐Tau N368 with calretinin (f). Scale bars were as indicated in each image

To verify the role of the truncated hTau N368 accumulation, we first used the same strategy as described in Figure 1a to achieve a unilateral mossy cell‐specific overexpression of hTau N368 in C57BL/6 mice (Figure 2a). By immunofluorescence staining, we observed that the mossy cell accumulated hTau N368 exhibited the AD‐like pathological hyperphosphorylation and oligomerization assessed by using AT8 and T22 antibodies, which was not detected by overexpressing full‐length hTau for 1 month (Figure 2b–d). A prominent accumulation of the AD‐like pathologically truncated hTau N368 was also detected in hilus of 10‐month 3xTg‐AD mice, and most hTau N368‐positive cells in hilus were identified as mossy cells, as indicated by co‐labeling of anti‐hTau N368 with calretinin (Figure 2e–f). These data confirm a successful mossy cell‐specific overexpression of the truncated hTau N368, which induces more prominent AD‐like pathological tau accumulation in a short time period.

**FIGURE 3:**
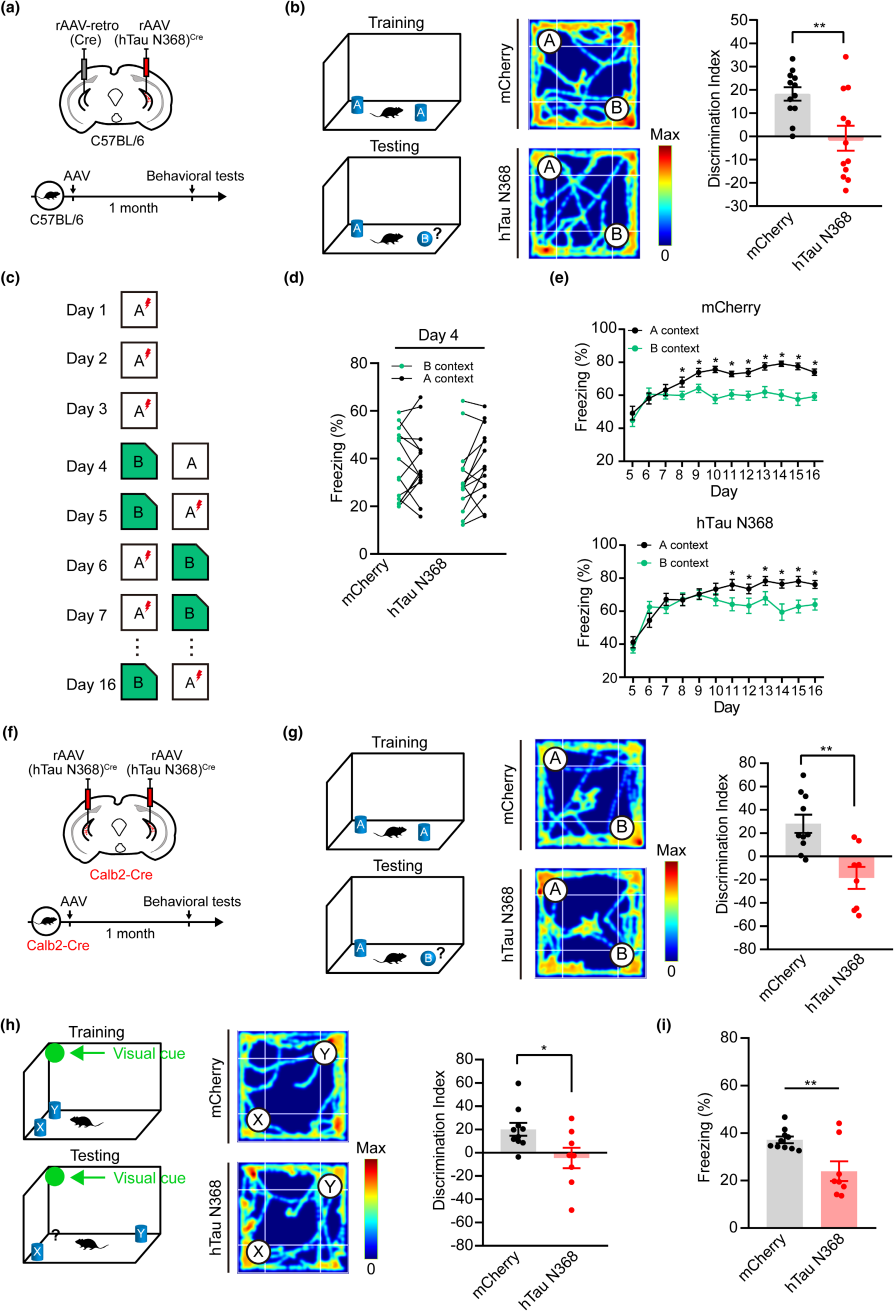
Both unilateral and bilateral mossy cell‐specific overexpressing hTau N368 for only 1 month induce cognitive impairments. (a, b) Mossy cell overexpressing hTau N368 in C57BL/6 mice for 1 month decreased discrimination index measured by object recognition test. The schematics showing experimental procedure (a), the behavioral paradigm in which the mice were tested to discriminate the novel object (object B) from the familiar object (object A) (b, left), the representative heatmaps showing the traveled traces of the mice during the test phase (b, middle) and the quantitative analysis (b, right). Unpaired *t* test, **p < 0.01, *n* = 12 mice. (c–e) Mossy cell overexpressing hTau N368 in C57BL/6 mice for 1 month decreased the ability in pattern separation. (c) Schematics showing procedures of contextual acquisition and pattern separation. In this paradigm, each mouse was trained to acquire fear memory by foot shock delivered in context A at days 1 to 3, and then tested to discriminate a similar context (context B) from A at day 4. In the subsequent days, the mice were trained and tested to discriminate a pair of similar contexts. (d) At day 4, both control and hTau N368‐overexpressing mice could not distinguish the two similar contexts. Paired *t* test, *p < 0.05, *n* = 14 mice. (e) At days 5 to 16, both control and the hTau N368‐overexpressing mice could learn to discriminate the context B from A, but the control group learned much faster than the hTau N368‐overexpressing group. Repeated‐measure ANOVA followed by Tukey's multiple comparisons tests, or paired *t* test. *p < 0.05, **p < 0.01, ***p < 0.001, *n* = 14 mice. (f–i) Mossy cell overexpressing hTau N368 in Calb2‐Cre mice for 1 month decreased discrimination and fear memory of the mice. (f) Strategy for virus infusion in Calb2‐Cre mice: rAAV‐hTau N368^Cre^ was infused stereotaxically into both sides of the hippocampal DG and the expression of hTau N368 was confirmed by immunofluorescence staining after 1 month (see Figure S4), and then the behavioral tests were performed. (g, h) Mossy cell overexpressing hTau N368 for 1 month decreased discrimination index of the mice in the object recognition (g) and object location (h) tests. In this paradigm, mice were tested to discriminate the novel object (object B) from familiar object (object A) (g), or the object (object Y) moved to a novel place (h). Representative heatmaps showing the travel traces of the mice during the test phase. Unpaired *t* test, *p < 0.05, **p < 0.01, *n* = 8–10 mice. (i) Mossy cell overexpressing hTau N368 for 1 month impaired contextual fear memory shown by the decreased freezing time during fear conditioning retrieval tests. Unpaired *t* test, **p < 0.01. *n* = 8–10 mice. Data were represented as mean ± *SEM*

Behavioral tests were carried at 1 month after unilateral mossy cell‐specific overexpression of hTau N368 in C57BL/6 mice (Figure 3a). In ORT, the discrimination index was significantly lower in hTau N368 mice than the control group (Figure 3b), while no difference was shown during CFC test (Figure S3). Mossy cells are mainly involved in pattern separation (Nakazawa, 2017). Therefore, we tested the pattern separation of hTau N368 mice. On the first 3 days, the mice were placed only into context A and received a foot‐shock after 180 s, and the freezing levels were assessed for the first 3 min before shock delivery. During the conditioning, the hTau N368 mice acquired similar level of fear memory to context A as the control mice. The mice visited context A and then context B on day 4. Then, the mice were subsequently trained to discriminate these contexts by visiting the two contexts daily for 12 days, receiving a foot‐shock 180 s after being placed in context A but not context B (Figure 3c). Quantitative analysis data showed that the control mice exhibited significant discriminating ability after day 8, whereas the mice with hTau N368 overexpression could not efficiently discriminate the contexts until day 11 (Figure 3d–e), suggesting learning and memory deficits.

To achieve a bilateral mossy cell‐specific overexpression of hTau N368, we used Calb2‐Cre mice, a transgenic line in which the Cre recombinase is specifically expressed in the ventral hilar mossy cells (Blasco‐Ibanez & Freund, 1997; Cembrowski et al., 2016; Fujise et al., 1998; Taniguchi et al., 2011). AAV‐EF1α‐DIO‐hTau N368‐mCherry (the hTau N368 group) or AAV‐EF1α‐DIO‐mCherry (the mCherry group) was infused into the DG subset of Calb2‐Cre mice (Figure 3f). Co‐labeling of mCherry or Tau N368 with calretinin but not with interneuron markers was detected bilaterally in vDG subset (Figure S4). One month after mossy cell infusion of hTau N368, the mice with hTau N368 overexpression showed a significantly decreased discrimination index in ORT (Figure 3g). In the OLT, hTau N368 mice were unable to distinguish the moved object from the unmoved object, indicating spatial memory deficit in hTau N368 mice (Figure 3h). In the CFC test, the hTau N368 mice showed less freezing time in retrial session, indicating an impaired contextual fear memory in hTau N368 mice (Figure 3i).

These data together demonstrate that both unilateral and bilateral mossy cell‐specific overexpressing hTau N368 for only 1 month could induce prominent cognitive impairment in mice.

### 2.4 Mossy cell‐specific overexpressing hTau N368 inhibits local excitatory neuronal activity with suppressed local neural network activity

**FIGURE 4:**
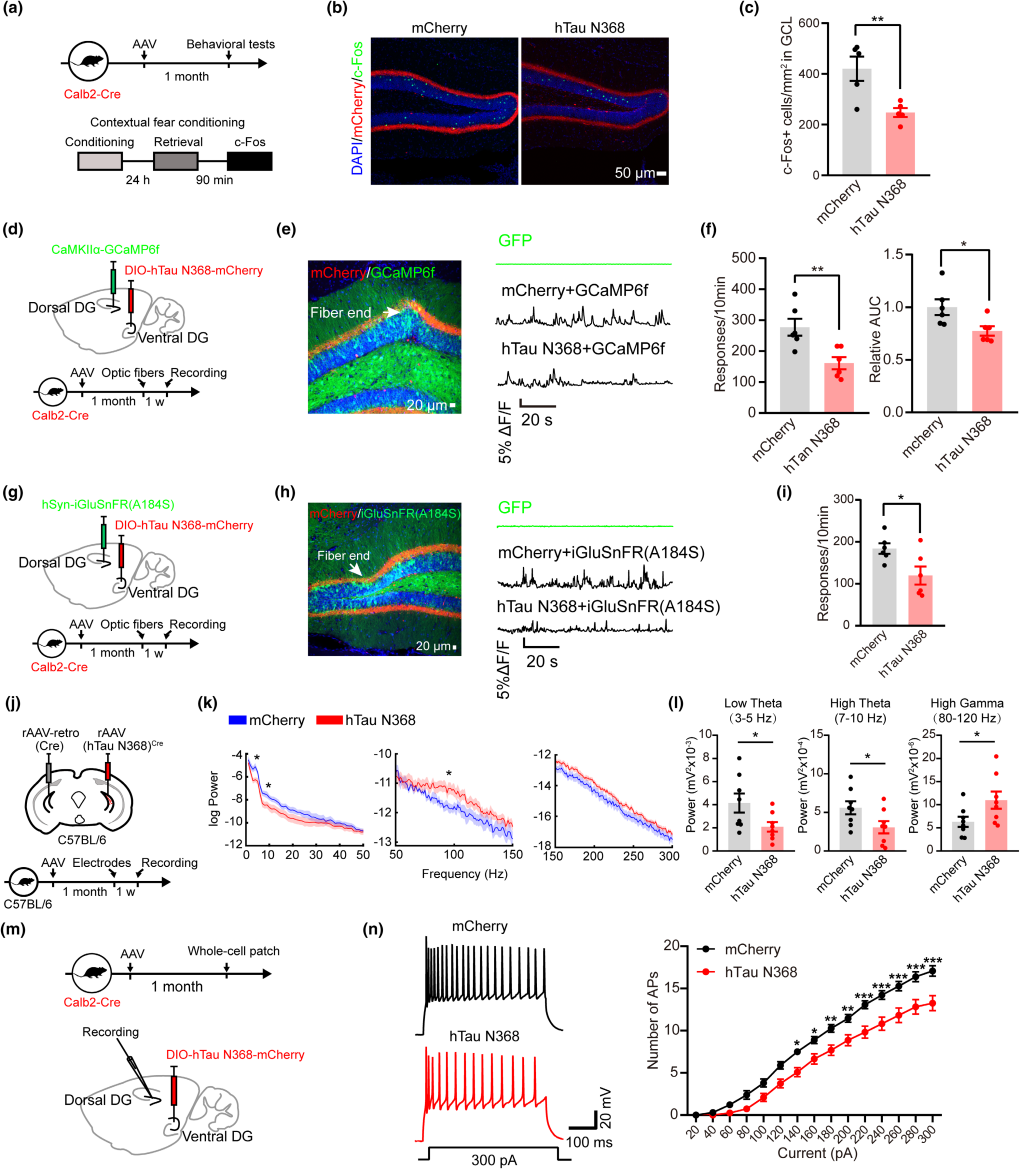
Mossy cell‐specific overexpressing hTau N368 inhibits local excitatory neuronal activity with suppressed local neural network activity. (a–c) Mossy cell‐specific overexpressing hTau N368 in Calb2‐Cre mice for 1 month inhibited neural activity shown by the significantly decreased numbers of c‐Fos+cells in granule cell layer (GCL) measured at 90 min after contextual fear conditioning test. The schematics of the experimental procedure (a), representative co‐immunofluorescence images (b) and the quantitative analysis (c). Unpaired *t* tests, *n* = 5 mice in each group, **p < 0.01. Scale bar, 50 μm. (d–f) Mossy cell overexpressing hTau N368 in Calb2‐Cre mice decreased calcium response in DG excitatory neurons evidenced by the decreased number of calcium response (f, left) and the area under Δ*F*/*F* curves (AUC) (f, right). The AAV‐carried hTau N368 and GCaMP6f were respectively infused into the ventral and dorsal DG subsets of Calb2‐Cre mice for 1 month, then the optic fibers were implanted and in vivo optic fiber recording was carried after 1 week. The schematics (d), representative image and Δ*F*/*F* signals presented by using 5% Δ*F*/*F* as threshold (e) and quantitative analysis (f). Scale bar, 20 μm. Unpaired *t* tests, *n* = 6 mice in each group, *p < 0.05, **p < 0.01. (g–i) Mossy cell‐specific overexpressing hTau N368 suppressed glutamate response in dDG evidenced by the decreased Δ*F*/*F* signals of iGluSnFR(A184S). The AAV‐carried hTau N368 and iGluSnFR(A184S) were respectively infused into the ventral and dorsal DG subsets of Calb2‐Cre mice for 1 month, then the optic fibers were implanted and the in vivo optic fiber recording was carried after 1 week. The schematic of the experimental procedure (g), the representative image and Δ*F*/*F* signal presented by using 5% Δ*F*/*F* as threshold (h) and the quantitative analysis (i). Scale bar, 20 μm. Unpaired *t* tests, *n* = 6 mice in each group, *p < 0.05. (j–l) Mossy cell overexpressing hTau N368 in C57BL/6 mice impaired local field potential (LFP) with reduced power spectral density (PSD) in dDG subset measured by in vivo electrophysiology. The schematic of the experimental procedure (j), PSD of LFP from 0.5 to 300 Hz (k), LFP band‐power in the low theta (3–5 Hz), high theta (7–10 Hz), and high gamma (80–120 Hz) (l). *n* = 8 mice, unpaired *t* tests, *p < 0.05. (m, n) Mossy cell‐specific overexpressing hTau N368 suppressed dDG granule cells excitability measured by whole patch clamp recording. The schematics (m), representative traces (n, left) and summary graphs (n, right) of intrinsic excitability of dDG granule cells measured as number of action potential (AP) in response to stepwise depolarizing currents (duration 500 ms) in DG slices. mCherry, *n* = 26 cells/5 mice; hTau N368, *n* = 23 cells/5 mice. Two‐way ANOVA, *p < 0.05, **p < 0.01, ***p < 0.001. Data were represented as mean ± *SEM*

To explore the influence of mossy cell‐overexpressing hTau N368 on the excitatory neuron activity, we infused AAV‐EF1α‐DIO‐hTau N368‐mCherry into the vDG of Calb2‐Cre mice for 1 month and measured the numbers of c‐Fos+granule cells at 90 min after CFC test (Figure 4a). The results showed that bilateral vDG mossy cell‐specific overexpressing hTau N368 for 1 month significantly decreased the numbers of c‐Fos+granule cells (Figure 4b,c). By simultaneously infused AAV‐EF1α‐DIO‐hTau N368‐mCherry to the vDG and AAV‐CaMKIIα‐GCaMP6f to the dDG of Calb2‐Cre mice, we measured the local excitatory neuron activity by calcium response (Figure 4d). We observed that bilateral mossy cell‐specific overexpressing hTau N368 significantly suppressed calcium response compared with mice infected with AAV‐mCherry or AAV‐EGFP empty vector (Figure 4e,f). The remarkably suppressed calcium response was also detected by ipsilaterally expressing hTau N368 in vDG mossy cells and AAV‐CaMKIIα‐GCaMP6f in dDG of C57BL/6 mice (Figure S5). These data together indicate that mossy cell overexpression of hTau N368 inhibits local excitatory neuron activity.

To further verify whether the observed hTau N368‐induced local excitatory inhibition was a result of impairment in glutamate transmission, we directly measured glutamate response in vivo with iGluSnFR(A184S) (Marvin et al., 2018), a genetically engineered glutamate sensor iGluSnFR(A184S) and hTau N368 were respectively infused into dorsal and vDG subsets of Calb2‐Cre mice for 1 month (Figure 4g). The representative image confirmed the virus infection (Figure 4h). The mice with mossy cell hTau N368 overexpression showed significantly suppressed glutamate responses (taken 5% Δ*F*/*F* as the threshold) in the dDG evidenced by the decreased Δ*F*/*F* signals of iGluSnFR(A184S) compared with the control mice (Figure 4h,i). These data confirm the excitatory neuron impairment by mossy cell‐specific hTau N368 accumulation.

Brain rhythms are periodically fluctuating waves of neuronal activity that are readily observed using local field potential (LFP) recording (Colgin, 2016), and theta frequency fluctuations of the LFP have long been implicated in learning and memory (Herweg et al., 2020). To explore the mechanisms underlying mossy cell‐hTau N368‐induced cognitive deficits, we used in vivo electrophysiology to record the LFP in the dDG subset of C57BL/6 mice. The microelectrodes were implanted into dDG of mice after vDG mossy cell‐overexpressing hTau N368 (Figure 4j). By analyzing the relative power of oscillations, we observed significantly decreased power at low‐theta (3–5 Hz) and high‐theta (7–10 Hz) oscillations with an increased high‐gamma (80–120 Hz) in the hTau N368 mice compared with the control mice (Figure 4k,l). By patch clamp recording on the brain slices, we observed that bilateral vDG mossy cell‐specific overexpressing hTau N368 remarkably suppressed the excitability of dDG granule cells (Figure 4m,n). These data together demonstrate that mossy cell‐specific hTau N368 accumulation induces dDG neural network dysfunction.

We also detected AHN. The results showed that mossy cell‐specific overexpressing hTau N368 did not change the number of BrdU‐, DCX‐, and NeuroD1‐immunoreactive cells (Figure S7), suggesting that mossy cell hTau N368 accumulation does not affect AHN which is significantly different from those observed in GABAergic neuronal hTau accumulation (Zheng et al., 2020).

### 2.5 Mossy cell‐specific overexpressing hTau N368 decreases multiple synapse‐associated proteins with disrupted Jak‐STAT and AKT‐GSK‐3β signaling pathway

**FIGURE 5:**
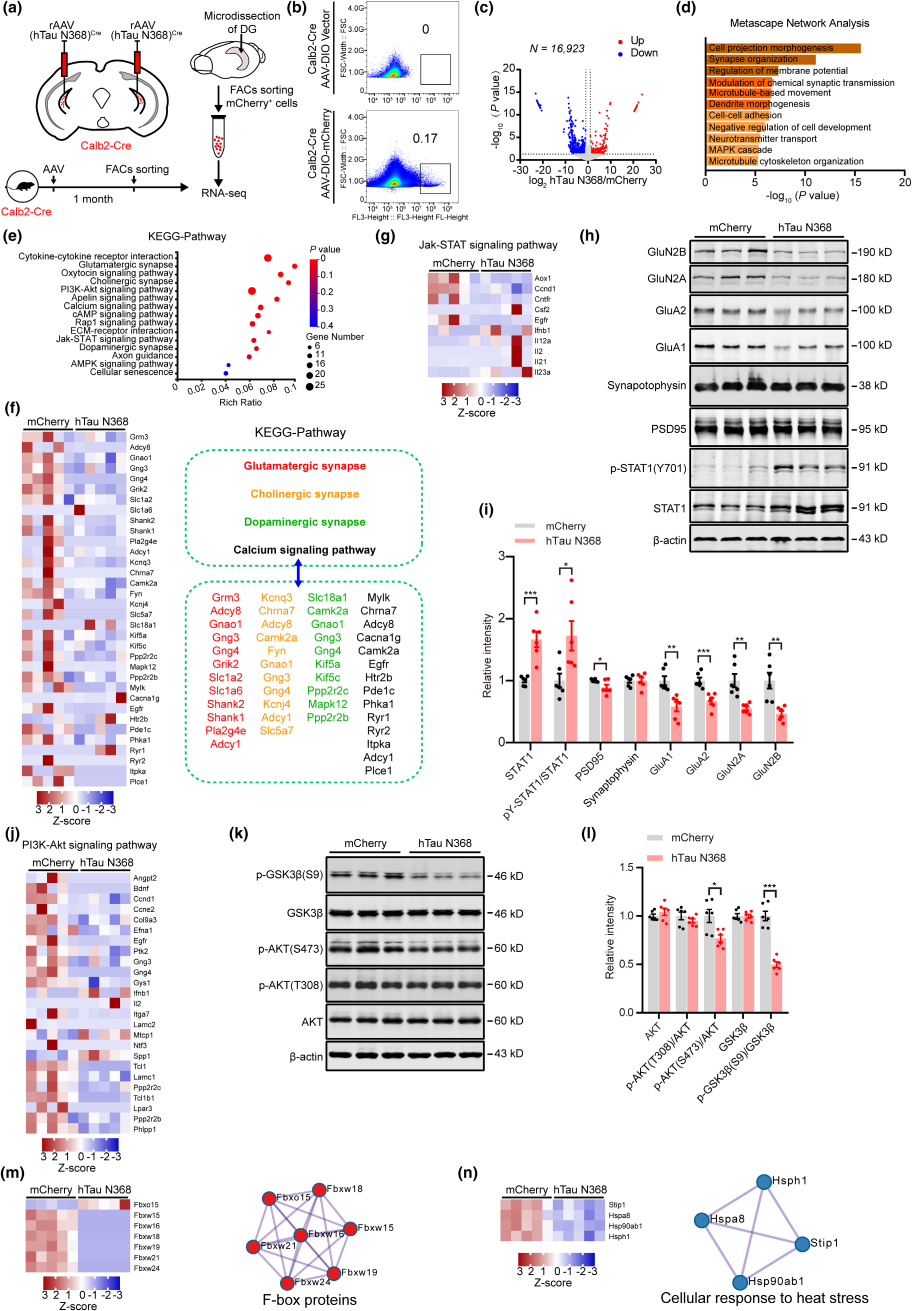
Mossy cell‐specific overexpressing hTau N368 decrease multiple synapse‐associated proteins with remarkably disrupted Jak‐STAT and PI3K‐AKT signaling pathway. (a) Schematics show sample preparation for transcriptomic analysis. The mossy cell‐specific overexpressing hTau N368 in Calb2‐Cre mice was carried out as described in Figure S4. After 1 month, the DG subset was dissected and the hTau N368‐overexpressing mossy cells were sorted by fluorescence activated cell sorting (FACs) for transcriptomic analysis. (b) The mCherry‐positive cells were sorted by FACs, to evaluate the specificity of AAV‐delivered mCherry‐fused hTau N368 and mCherry expression in mossy cells. The boxed area (b, bottom panel) represents cells gated as significantly fluorescent compared with non‐fluorescent control (b, top panel). (c) A total of 917 in 16,923 genes were statistically significant up‐ or downregulated in mossy cells after hTau N368 overexpression measured by RNA‐seq analysis. *n* = 5 mice in each group. (d) Top Gene Ontology terms for differentially expressed genes (DEGs) in (c). (e) Enriched KEGG pathway of DEGs in (c). (f) Heatmap showing gene expression of DEGs in glutamatergic synapse, cholinergic synapse, dopaminergic synapse, and calcium signaling pathway. (g–i) Mossy cell overexpressing hTau N368 upregulated STAT1 with downregulated synapse‐associated proteins in dentate gyrus. Data were normalized to β‐actin and the mean value of mChery group for each protein. Unpaired *t* tests, *n* = 6 mice, *p < 0.05, **p < 0.01, ***p < 0.001. (j–l) Mossy cell overexpressing hTau N368 inhibited p‐AKT(S473) with reduced pGSK‐3β(S9) (indicating GSK3β activation). Data were normalized to β‐actin and the mean value of mChery group for each protein. Unpaired *t* tests, *n* = 6 mice, *p < 0.05, **p < 0.01, ***p < 0.001. (m, n) Heatmap showing gene expression of DEGs in F‐box proteins and cellular response to heat stress. Data were represented as mean ± *SEM*

To explore the molecular mechanisms underlying mossy cell‐hTau N368‐induced cognitive deficits, we carried out mossy cell RNA‐Seq analysis after mossy cell‐specific overexpressing hTau N368 in Calb2‐Cre mice. The mice were treated as indicated in Figure 3f and Figure S4 for 1 month, then the DG subset was dissected (Figure 5a), and the mCherry‐positive mossy cells (mCherry or mCherry‐fused hTau N368) were collected by FACs for RNA‐Seq analysis (Figure 5b). A total of 917 differentially expressed genes (DEGs) were identified, in which 190 were upregulated and 727 were downregulated (Figure 5c). Metascape Network Analysis for DEGs revealed several significantly enriched pathways, including cell projection morphogenesis, synapse organization, regulation of membrane, and modulation of chemical synaptic transmission (Figure 5d). Meanwhile, Kyoto Encyclopedia of Genes and Genomes (KEGG) pathway analysis on the RNA‐Seq data revealed that 54 pathways were significantly altered in the hTau N368 overexpression mossy cells (Figure 5e). Among these pathways, the genes on glutamatergic synapse were significantly decreased (Figure 5f), indicating a preferential impairment of hTau N368 on the synapse‐associated signalings.

A significant increase of STAT1 and pY‐STAT1(Y701) and reduced synaptic proteins, including PSD95, GluA1, GluA2, GluN2A, and GluN2B (Figure 5g–i), with decreased phosphor‐AKT at Ser473 and phosphor‐GSK‐3β at Ser9 (Figure 5j–l), were also detected in DG subset of the mossy cell‐specific hTau N368 overexpressing mice measured by KEGG pathway analysis and Western blotting. These data together indicate that inhibited expression of synaptic protein and activated JaK‐STAT1 and GSK‐3β could be the cause for memory loss by disrupting synaptic functions.

### 2.6 Chemogenetic activating mossy cell rescues hTau N368‐induced local network inhibition and cognitive impairments in mice

**FIGURE 6:**
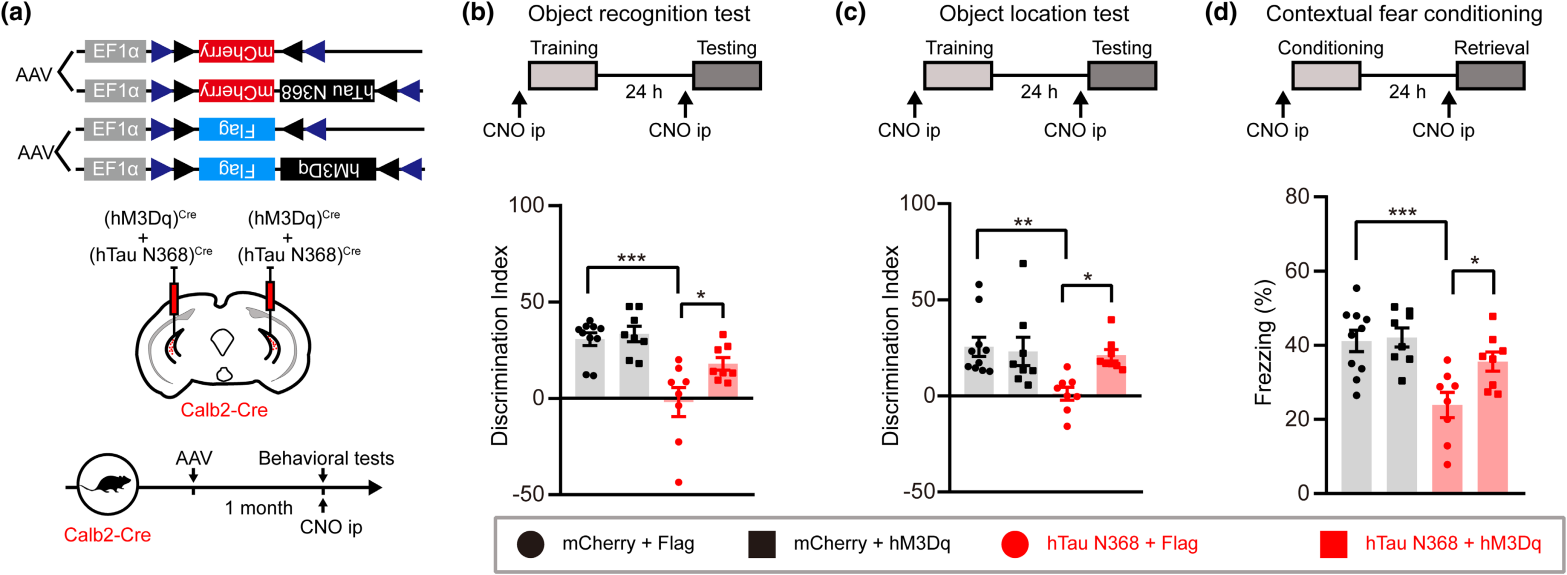
Chemogenetic activating mossy cells rescues hTau N368‐induced cognitive impairments in mice. (a–d) Mossy cell‐specific overexpressing hTau N368 in Calb2‐Cre mice for 1 month significantly decreased discrimination index and decreased freezing time measured respectively by novel object recognition test (b), object location test (c), and contexture fear conditioning (d), whereas chemogenetically activating mossy cells by intraperitoneal (ip) injecting CNO attenuated hTau N368‐induced cognitive deficits. Two‐way ANOVA followed by Tukey's multiple comparisons test, *n* = 8–10 mice, *p < 0.05, **p < 0.01, ***p < 0.001. Data were represented as mean ± *SEM*

To test whether strengthening glutamate transmission can rescue the cognition deficits induced by mossy cell hTau N368 overexpression, we co‐infused the Calb2‐Cre mice with DIO‐hM3Dq‐Flag or DIO‐Flag and DIO‐EF1α‐mCherry or DIO‐EF1α‐hTau N368‐mCherry, performed behavioral tests after 1 month (Figure 6a). In ORT, an increased discrimination index was shown in mice administrated with CNO with co‐expressing hTau N368+hM3Dq compared to the mice with co‐expressing hTau N368+Flag (Figure 6b). In OLT, chemogenetic activating mossy cells restored the ability to distinguish the displaced object in hTau N368 mice (Figure 6c). In CFC test, the hTau N368+hM3Dq mice showed more freezing time than the hTau N368+Flag group (Figure 6d). These data indicate that stimulating mossy cells could rescue the hTau N368‐induced local neural network inhibition and improve cognitive deficits in mice.

## 3 DISCUSSION

In the present study, we found a prominent accumulation of the hyperphosphorylated tau protein in DG mossy cells of 3xTg‐AD mice, in which accumulation of the pathologically truncated hTau N368 was correlated to the cognitive deficits. By using transgenic mouse (Taniguchi et al., 2011) and viral infection (Ratzliff et al., 2004) to label mossy cells, we successfully achieved unilateral and bilateral mossy cell‐specific overexpression of full‐length wild‐type hTau and hTau N368 in vDG subset. We found that mice gradually developed cognitive impairments with the mossy cell hTau accumulating, whereas the mice developed more severe pathologies and a more rapid cognitive deficit when mossy cell overexpressing the AD‐like pathologically truncated hTau N368. The mossy cell accumulation of hTau N368 resulted in dysregulation of multiple signaling pathways, leading to a decreased neural activity with an inhibited granule cell excitation and a suppressed ventral mossy cell to dorsal granule cell transmission, which eventually causes cognitive impairments as seen in the AD patients. Importantly, chemogenetic activation of mossy cells to strengthen glutamatergic transmission could efficiently rescue the hTau N368‐induced cognitive impairments in mice. These findings not only reveal new mechanisms underlying hTau‐induced cognitive deficits but also provide potential molecular targets and better experimental models for studying tauopathies, including AD.

Previous studies showed that an increased tau was associated with anxiety (Goncalves et al., 2020). Depression and anxiety are characteristic of AD patients and the anxiety behavior shown in 3xTg‐AD mice was age‐ and sex‐dependent (Kosel et al., 2020). In the current study, we did not detect significant anxiety‐like behavior after mossy cell overexpressing hTau N368. Many reasons could cause or contribute to the discrepancy, such as the differences in animal models, that is, hTau 368 overexpressed in vDG subset versus 3xTg‐AD (Oddo et al., 2003), the accumulating time of the pathologic gene, only 3 months observed here, and the sex of the animal, only male mice used here, etc.

The hippocampal DG has a key role in spatial memory formation (Hainmueller & Bartos, 2020), and many hypotheses have been made to depict how the architectural elements and various cell types in the DG may underlie its function in regulating cognitive functions. In AD patients, hippocampus is the region affected by tau pathology at early stage of the disease process (Thal et al., 2000). We recently reported that tau accumulation in DG GABAergic neurons induced local network hyperactivation and deficits of AHN, leading to astrogliosis in mice (Zheng et al., 2020). Here, we found that hTau accumulation in mossy cells could also induce spatial cognitive deficit with mechanisms involving the influence of hTau on the glutamatergic mossy cells themselves and the influence of the affected mossy cells on the local network activity. The current literature reports regarding the effects of mossy cells on granule cells were controversial, some show that mossy cell inhibition or ablation activates granule cells, whereas others report that ablation of mossy cells does not affect granule cells (Botterill et al., 2019; Jinde et al., 2012, 2013; Ratzliff et al., 2004; Wang et al., 2021). Furthermore, mossy cells can directly activate the distant granule cells or indirectly inhibit the granule cells through activating the local GABAergic neurons; thus, the final influence of mossy cells to granule cells should depend on which mechanism is dominant or what experimental conditions were employed. We overexpressed hTau for at least 1 month, by which we observed mossy cell inhibition with a suppressed dDG excitatory neuronal activity.

By in vivo optic fiber recording, we observed that the dDG glutamate response measured by iGluSnFR(A184S) was decreased. The iGluSnFR(A184S) signal contains the signals from dDG granule cells, interneurons, and mossy cells. Granule cells are the principal neurons in DG subset, which receive two powerful excitatory inputs, that is, the perforant path originated from the EC and the associational pathway originated from mossy cells, the principal neurons in hilus of DG (Hainmueller & Bartos, 2020; Kleschevnikov & Routtenberg, 2003; Woods et al., 2018). Thus, the decreased iGluSnFR(A184S) signal could be due to the reduced glutamate release from vDG or dDG mossy cells, and the excitatory inputs from EC. As we specifically expressed hTau N368 in vDG mossy cells, we speculate that the decreased iGluSnFR(A184S) signal should be mainly due to the reduced glutamate release of vDG mossy cells. Our data suggest that the mossy cell hTau N368 accumulation not only impairs its synaptic connections with granule cells but also alters its synaptic connections with GABAergic interneurons, the latter deserves further investigation. We also observed that the mice with mossy cell hTau N368 overexpression were showed a decreased theta power, while the high‐gamma power was increased. The fluctuation of theta frequency has long been implicated in learning and memory (Colgin, 2013). Recent studies also suggest that high gamma is linked to working memory or encoding of current sensory information in memory (Bieri et al., 2014; Yamamoto et al., 2014; Zheng et al., 2016), but conflict results have been shown.

The exact function for different hippocampal sub‐regions is also controversial. Currently, it is believed that the dorsal hippocampus is engaged in spatial navigation and memory, while the ventral hippocampus is responsible for anxiety‐related behaviors (Strange et al., 2014). The mossy cells in DG project their processes both ipsilaterally and contralaterally. The ventral and dorsal mossy cells have different projection characteristics, that is, the ventral mossy cells mainly project to the inner‐molecular layer (IML) of the DG, whereas the dorsal mossy cells project to both inner‐ and meso‐molecular layers (Botterill, Gerencer, et al., 2021; Houser et al., 2021). The bodies of granule cells form the granule cell layer, and their dendrites are present in the molecular layer (Amaral et al., 2007). The electron microscopy showed that the terminals of vDG mossy cells distant projection primarily innervate spines in the IML (Buckmaster et al., 1996). Since interneurons with dendrites in the IML rarely have spines, it is likely that the distant mossy cell axon projection primarily innervates granule cells (Buckmaster et al., 1996). As the number of ventral mossy cells overwhelms that of the dorsal, both methods we used to achieve mossy cell‐specific overexpression of hTau or hTau N368 were mainly on the ventral subset of DG in our current study. We mainly detected the alterations of dorsal excitatory neuronal activity after ventral mossy cell‐specific overexpression of hTau N368. An inhibited local neuronal network excitation in dDG with the suppressed local excitatory neuronal activity in dDG were shown. Previous studies also showed that the cleaved hTau fragment, that is, hTau N368 produced during aging or AD, is more toxic than the full‐length hTau (Shimonaka et al., 2020; Xiang et al., 2019; Zhang et al., 2014, 2021). We also found in the current study that mossy cell‐specific overexpressing hTau N368 induced a more severe accumulation of the pathological tau and a more rapidly appeared cognitive deficit than the full‐length hTau. Currently, no gene mutation on tau molecule has been identified in AD patients. To establish the AD‐like experimental models, scientists have to use mutated tau, such as P301S or P301L. Strictly speaking, these experimental models do not represent AD. Therefore, our results suggest that overexpressing hTau N368 may serve as a better model for tauopathies, including the sporadic AD which accounts for over 95% of the AD cases.

The molecular mechanism underlying hTau‐induced neurodegeneration and cognitive deficit is still not fully understood, and no report has been found regarding the effect of hTau on mossy cells. By using fluorescence‐activated cell sorting, we prepared purified mossy cells with hTau N368 overexpression and made the transcriptomic analysis by RNA‐seq. We found that mossy cell‐hTau N368 accumulating affected multiple signaling pathways associated with a deregulated synapse function, which may contribute to synapse dysfunction. More interestingly, the gene expression of several F‐box proteins and heat‐shock proteins were significantly reduced after mossy cell overexpression of hTau N368 (Figure 5m,n). F‐box proteins function as the substrate recognition subunits of S‐phase kinase‐associated protein 1 (SKP1)−Cullin1 (CUL1)−F‐box protein (SCF) ubiquitin ligase complexes (Ho et al., 2006; Kipreos & Pagano, 2000). We speculate that these downregulated F‐box proteins may impair ubiquitin‐proteasome system and thus lead to neuronal dysfunction, which deserves further investigation. Heat‐shock proteins (HSPs) play a central role in regulating protein quality control and contribute to protein aggregation and disaggregation (Campanella et al., 2018; Kampinga & Bergink, 2016). Here, we found that the gene expression of HSP90ab1 was significantly decreased after mossy cell overexpression of hTau N368; thus, HSP90ab1 could be a potential molecular target for tauopathies.

Synaptic failure strongly correlates with cognitive decline. We also observed that the proteins levels of PSD95, GluA1, GluA2, GluN2A, and GluN2B were significantly decreased after mossy cells overexpression of hTau N368, which could be the direct cause for the impaired synaptic function in DG by hTau N368 accumulation. We also observed AKT activity was inhibited and GSK‐3β was activated. GSK‐3β is activated in the brain of AD patient and the AD mouse model, and activation of GSK‐3β induces tau hyperphosphorylation and consequently neurodegeneration. Thus, hTau N368 overexpression in mossy cells induces dysregulation of AKT/GSK3β, which in turn causes a persistent tau hyperphosphorylation, forming a vicious circle.

In conclusion, hTau accumulation inactivates mossy cells in DG, which in turn causes inhibition of local neural network activity, leading to hippocampus‐dependent spatial cognitive deficits, stimulating mossy cells attenuates hTau‐induced impairments.

## 4 EXPERIMENTAL PROCEDURES

See the Appendix S1.

## CONFLICT OF INTEREST

The authors declare no competing interests.

## AUTHOR CONTRIBUTIONS

J‐ZW, EL, and SL designed research; SL, QZ, NY, HD, and YW performed the experiments; SL, QZ, and J‐ZW analyzed data; SL and J‐ZW wrote the manuscript; HY, WW, ML, YG, GP, XW, and DK helped with the experiments and made constructive suggestion.

## Supporting information

Appendix S1

Figure S1‐S8

## Data Availability

The data used to support the findings of this study are available from the corresponding author upon request.
