# Supplementary material for: Alzheimer‐like tau accumulation in dentate gyrus mossy cells induces spatial cognitive deficits by disrupting multiple memory‐related signaling and inhibiting local neural circuit
Source: Aging Cell. 2022 Mar 31;21(5):e13600. doi: 10.1111/acel.13600 (PMC9124302; doi:10.1111/acel.13600)
Supplement: Supplementary file 1 — Appendix S1 [file ACEL-21-e13600-s002.docx]

**Supplemental Information**

**Materials and methods**

**1.1** | **Animals**

C57BL/6 mice were purchased from Vital River Laboratories (Beijing, China). Calb2-Cre (B6(Cg)-Calb2^tm1(cre)Zjh^/J) mice were generous gifts of prof. Kexin Yuan (Tsinghua University, Beijing, China). 3xTg-AD (129S4.Cg-Tg(APPSwe,tauP301L)1Lfa Psen1^tm1Mpm^/LfaJ) mice and wild-type 129 (129S1/SvImJ) mice were purchased from Jackson Laboratory. All mice were kept under standard laboratory conditions, with a 12 h alternating light/dark cycle, food and water available ad libitum. Except specifically stated in the text, only male mice (8-12 weeks, weighing 20-30 g) were used for virus injection. Brain sections from 7-month and 10-month 3xTg-AD and age-matched wild-type mice were used for phosphor-tau and anti-Tau N368 immunostaining. All animal experiments were approved by the Animal Care and Use Committee of Huazhong University of Science and Technology.

**1.2** | **Stereotactic injection**

Mice (20-30 g) were anesthetized with 1% pentobarbital sodium (35 mg/kg) and placed into a stereotactic apparatus (RWD, Shenzhen, China). For hTau or hTau N368 overexpression in C57BL/6 mossy cells, a retrograde variant of recombinant adeno-associated virus (rAAV-retro; 3.0 mm posterior, 2.2 mm left, 2.5 mm ventral to bregma) expressing Cre-recombinase were injected into the contralateral dentate gyrus, and virus encoding a Cre-dependent mCherry (AAV-EF1α-DIO-mCherry; 3.0 mm posterior, 2.2 mm right, 2.5 mm ventral to bregma), hTau (AAV-EF1α-DIO-hTau-mCherry; 3.0 mm posterior, 2.2 mm right, 2.5 mm ventral to bregma) or hTau N368 (AAV-EF1α-DIO-hTau N368-mCherry; 3.0 mm posterior, 2.2 mm right, 2.5 mm ventral to bregma) were injected ipsilaterally into the hilar of dentate gyrus. For calcium imaging recording in C57BL/6 mice, GcaMP6f (AAV-CaMKllα-GcaMP6f or AAV-CaMKllα-GFP; 2.0 mm posterior, 1.5 mm right, 2.0 mm ventral to bregma) were injected to the ipsilaterally of mossy cells hTau N368 or mCherry overexpression. Calb2-Cre mice were injected with the Cre-dependent virus mCherry (AAV-EF1α-DIO-mCherry; 3.0 mm posterior, 2.2 mm left and right, 2.5 mm ventral to bregma) or hTau N368 (AAV-EF1α-DIO-hTau N368-mCherry; 3.0 mm posterior, 2.2 mm left and right, 2.5 mm ventral to bregma). For calcium imaging recording in Calb2-Cre mice, GcaMP6f (AAV-CaMKllα-GcaMP6f or AAV-CaMKllα-GFP; 2.0 mm posterior, 1.5 mm left and or/right, 2.0 mm ventral to bregma) were injected to the dentate gyrus, meanwhile mCherry or hTau N368 were overexpression in mossy cells. The glutamate sensor iGluSnFR(A184S) (AAV-hSyn-iGluSnFR(A184S) or AAV-hSyn-GFP; 2.0 mm posterior, 1.5 mm left and or/right, 2.0 mm ventral to bregma) were injected to Calb2-Cre mice, meanwhile mCherry or hTau N368 were overexpression in mossy cells. For Chemogenetic activation of mossy cells, mCherry group mice were co-injected with AAV-EF1α-DIO-hM3Dq-flag or AAV-EF1α-DIO-Flag, hTau N368 group mice were co-injected with AAV-EF1α-DIO-hM3Dq-flag or AAV-EF1α-DIO-Flag. The titer of AAVs ranges from 2.5E+12 to 6.8E+12 vg/mL. The virus was delivered via a 2 µl syringe and a 25 gauge metal needle; the injection volume and flow rate were 0.5 µl at 0.1 µl/min. The needle was left in place after injection for 5 min before slowly being withdrawn. The skin was then sutured and sterilized with iodophors. After the virus infection, the location of the virus was confirmed by examining the virus-expressed mCherry, GFP, respectively. Subjects with off-target virus infusion were excluded from analysis.

**1.3** | **Immunostaining**

For animal experiments, mice were anesthetized with 1% pentobarbital sodium and intracardially perfused with PBS followed by 4% paraformaldehyde solution (PFA, in 0.01 M PBS, pH 7.4). The brains were removed from the skull, left in the 4% PFA for 12 h post-fixation, and then cryoprotected in 20% sucrose/PBS and 30% sucrose/PBS solutions in turn. Brain sections were sliced with cryostat microtome (CM1900, Leica) at 50 μm thickness. The free-floating brain section were collected in PBS. For immunofluorescence staining, free-floating brain sections were first washed with PBS, then blocked with 5% bull serum albumin/0.5% Triton X-100/PBS for 30 min, and then incubated with primary antibodies in 3% bull serum albumin/0.3% Triton X-100/PBS at 4°C for 24 h. After three PBS washes, sections were incubated with secondary antibodies at 37°C for 1 h (dilution in 0.3% PBST for all antibodies), and finally mounted onto slides with 50% glycerin-PBS (vol/vol) solution. Specifically, for BrdU staining, sections were treated with 2 N HCl for 30 min at 37°C followed by 10 min washes in 0.1 M sodium borate (pH 8.5) before blocking and antibodies incubation. For immunohistochemistry staining, free-floating sections were incubated with 0.3% H_2_O_2_ (in PBS) at 37°C for 30 min to eliminate endogenous peroxidase activity before serum blocking. Brain sections were developed using a DAB peroxidase substrate kit (ZSGB-BIO). Sections were then dehydrated by graded ethanol and xylene and fixed by balsam. Images were taken using an Olympus SV120 virtual slide Microscope or LSM710 confocal laser-scanning microscope (Zeiss). The following antibodies were used: anti-Tau (Phospho-Thr205) (Signalway Antibody, 11108), anti-Tau (Phospho-Thr231) (Signalway Antibody, 11110), HT7 (ThermoFisher Scientific, MN1000), AT8 (ThermoFisher Scientific MN1020), anti-Tau (T22) (Sigma-Aldrich, ABN454-I), anti-Calretinin (Abcam, ab702), anti-Calretinin (Sigma-Aldrich, MAB1568), anti-Parvalbumin (Abcam, ab11427), anti-Somatostatin (ImmunoStar, 20067), anti-GAD67 (Sigma-Aldrich, MAB5406), anti-Tau N368 (a generous gift from Prof. Keqiang Ye, Emory University School of Medicine), anti-BrdU (Abcam, ab152095), anti-DCX (Cell Signaling, 4604S), anti-NeuroD1 (Abcam, ab213725), anti-c-Fos (Cell Signaling, 2250).

**1.4** | **Evaluation of adult hippocampal neurogenesis (AHN)**

In the present study, the dorsal DG was used for evaluation of AHN. A series of images was obtained by z-stack scanning at a 3 μm interval throughout the entire 50 μm-thickness. BrdU (B9285, Sigma-Aldrich) was reconstituted at 10 mg per ml in PBS solution and injected (intraperitoneally, 50 μg/g) for 5 consecutive days before execution for the evaluation of cell proliferation. Quantification of cell numbers was performed as previously described (Zheng et al., 2017). Briefly, every fifth and a total of 3 sections were stained and then counted by an experimenter blinded to experimental conditions. Counts were multiplied by 5 to calculate the total number of cells in the dorsal DG.

**1.5** | **Protein extraction and immunoblotting**

Mouse hippocampus dentate gyrus were microdissected as described (Hagihara et al., 2009), then homogenized in the lysis buffer [100 mM NaCl, 50 mM Tris·HCl pH 7.4-7.5, 5 mM EDTA, 1% (vol/vol) Triton X-100, 1:100 PMSF, 1:1,000 protease inhibitor cocktail containing 4-(2-Aminoethyl)-benzenesulfonyl fluride hydrochloride, aprotinin, bestatin, leupeptin, E-64, and pepstatin A stored at 4 °C] and then placed on ice for 30 min. Homogenates were centrifuged at 12,000 x g for 15 min at 4 °C. The supernatant was collected and the protein concentration was measured using the BCA assay (BCA, KF016, Sigma-Aldrich). Proteins were separated by SDS-PAGE gels and transferred to nitrocellulose membranes (Merck Millipore). Membranes were then blocked with 5% nonfat milk in TBS for 1 h at room temperature, followed by incubation with the primary antibody overnight at 4 °C. After washing, membranes were incubated with IRDyeⓇ-conjugated secondary antibodies (1:5,000 - 1:10,000 dilutions) for 1 h at room temperature. Blots were visualized using an Odyssey Imaging System (LI-COR Biosciences), and quantified using ImageJ (Fiji) software (Schindelin et al., 2012). The following antibodies were used: anti-β-actin (Abcam, ab6276), anti-STAT1 (Cell Signaling, 14994), anti-p-STAT1 (Tyr701) (Cell Signaling, 9167), anti-PSD95 (Cell Signaling, 3450), anti-Synaptophysin (Cell Signaling, 36406), anti-GluA1 (Cell Signaling, 13185), anti-GluA2 (Cell Signaling, 13607), anti-GluN2A (Cell Signaling, 4205), anti-GluN2B (Cell Signaling, 14544), anti-AKT (Cell Signaling, 9272), anti-p-AKT (Thr308) (Cell Signaling, 9275), anti-p-AKT (Ser473) (Cell Signaling, 4058), anti-GSK3β (Cell Signaling, 12456), anti-p-GSK3β (Ser9) (Cell Signaling, 14630).

**1.6** | ***In vivo* electrophysiology**

Mice were anesthetized with 1% pentobarbital sodium and head-fixed in a stereotaxic instrument. After the skull was exposed, three anchor screws were drilled into the skull. A small (approximately 2 x 2 mm) craniotomy was drilled on the right hemisphere. The dura was perforated using a needle. A custom designed recording microdrive, with 4 combined but movable tungsten tetrodes and two reference electrodes were implanted into the dentate gyrus (2.0 mm posterior, 1.5 mm right, 2.0 mm ventral to bregma), or cerebellum, respectively. The craniotomy and implant was sealed with dental cement (Medental, Beijing). Mice were housed individually housed in home cages. One week after the surgery, the microdrive was advanced gradually to lower the optetrode to the desired anatomical location (Bragin et al., 1995). Recordings then were performed in an enrichment environment. Following *in vivo* recordings, mice were perfused with 4% PFA. Mice were excluded if the location of the electrodes was off-target.

The data acquisition and analysis was performed as previously described (Du et al., 2016). All electrophysiological recordings were performed using the OmniPlexD Neural Data Acquisition System (Plexon Inc.). For LFP analysis, signals were down-sampled to a rate of 1,000 Hz and low-pass filtered at 300 Hz. Time-frequency decomposition of the LFP signal was performed using custom code in Matlab and Chronux, an open-source software package for the analysis of neural data.

**1.7** | ***In vitro* electrophysiology**

*In vitro* electrophysiology was performed as described previously (Ge et al., 2019). The mice were anesthetized with 1% pentobarbital sodium, and the brains were quickly removed and placed in chilled ice-cold artificial cerebrospinal fluid (ACSF) containing (in mM): 110 choline chloride, 1.3 NaH_2_PO_4_, 2.5 KCl, 25.0 NaHCO_3_, 0.5 CaCl_2_, 7 MgCl_2_, 20 glucose, 1.3 Na-ascorbate, 0.6 Na-pyruvate (oxygenated with 95% O_2_ and 5 % CO_2_). The coronal brain slices (300 μm thick) were prepared using a Leica VT1000S vibratome (Leica, Germany) and transferred to a holding chamber containing oxygenated ACSF, composed of (in mM) NaCl 125, NaH_2_PO_4_ 1.3, KCl 2.5, NaHCO_3_ 25, MgCl_2_ 1.3, CaCl_2_ 2, Na-ascorbate 1.3, Na-pyruvate 0.6, glucose 10, incubated at 34.5 °C for 30 minutes and at 25 ± 1 °C for additional 1 h. All solutions were saturated with 95% O_2_ / 5% CO_2_ (vol/vol). For recordings, slices were held in a small chamber superfused (2 ml/min) with oxygenated ACSF at room temperature. Cells were visualized with an upright microscope (Olympus, BX51WI) with infrared differential interference contrast (DIC) optics.

Action potentials were evoked by a series of current injections from 0 pA to 300 pA in 20 pA steps for 500 ms. A high K^+^ intracellular solution was filled into recording pipette (4-6 MΩ) containing (in mM): 140 potassium-gluconate, 5 KCl, 2 MgCl_2_, 0.2 EGTA, 10 HEPES, 4 MgATP, 0.3 NaGTP, 10 Na_2_ Phosphocreatine (pH 7.2, 290-300 mOsm).

Electrodes were filled with ACSF and had resistances of 4-6 MΩ. All data were recorded with an Axonpatch 700B amplifier (Molecular Devices) and a Digidata 1550B (Molecular Devices). Data were collected at 10 kHz, filtered with a low-pass filter at 2 kHz, and analyzed using ClampFit 10.2 software (Molecular Devices).

**1.8** | ***In vivo* optic fiber recording**

The optic fiber cannulas (NA = 0.37, Newdoon, China) implantation procedures were the same as tetrodes implantation described above. Each mouse was handled and adapted for 3 consecutive days in homecage before recording. GCaMP6f or iGluSnFR(A184S) signals were recorded using a fiber photometry system (Thinker Tech, China), with the LED power of 65 μW. The mice were allowed to explore freely in an enrichment environment during recordings. Data were analyzed using MATLAB, ΔF/F was calculated as: ΔF/F = (F - F_0_) / (F_0_ -F_offset_) x 100%. A threshold of 5% ΔF/F was set to count calcium or glutamate responses. Mice were excluded if the location of the optic fiber was off-target.

**1.9** | [**Fluorescence-activated**](javascript:;) [**cell**](javascript:;)**sorting (FACs)**

After Calb2-Cre mice mossy cell specifically overexpression hTau N368 for one month, the mice were killed by cervical dislocation and brains were quickly removed. Hippocampi dentate gyrus were isolated on ice as described (Hagihara et al., 2009), then dissociated in DMEM/F12 media, followed by digestion with 0.125% trypsin for 20 min at 37°C. Enzymatic activity was stopped by 10% FBS. Cell suspension was prepared by gentle trituration, and then centrifuged at 1000 rpm at 4°C for 10 min. The pellet was collected, resuspended and then filtered through a 0.45 μm membrane (Fisher Scientific) to obtain single-cell suspension in PBS. mCherry-positive cells were sorted through FACs (MOflo XDP, Beckman Coulter) and summit software. Cell suspension from empty vector (AAV-DIO Vector) carried Calb2-Cre mice hippocampi dentate gyrus was used as the non-fluorescent control.

**1.10** | **Total RNA extraction and mRNA library construction**

The mCherry-positive cells were sorted through FACs (MOflo XDP, Beckman Coulter), then used to RNA extraction. Total RNA from sorted cells was extracted using RNeasy Micro kit (QIAGEN, GER) according to the manual instruction. Total RNA was qualified and quantified using a Nano Drop and Agilent 2100 bioanalyzer (Thermo Fisher Scientific, MA, USA). High-quality total RNA was used for mRNA library construction.

Limited RNA (more than 200pg, high-quality) was amplified with oligo-dT and dNTPs, incubated at 72°C and immediately put back on ice, then reverse transcribed to cDNA based on polyA tail. The template was switched to the 5' end of the RNA and the full-length cDNA was generated by PCR. The average molecule length of PCR product was determined using Agilent 2100 bioanalyzer instrument (Thermo Fisher Scientific, MA, USA). The cDNA was purified and fragmented into small pieces with fragment buffer by PCR, and the product was purifiedand selected by the Agencourt AMPure XP-Medium kit (Thermo Fisher Scientific, USA). cDNA was quantified by Agilent Technologies 2100 bioanalyzer. The double stranded PCR product undergo QC step was heat denatured and circularized by the splint oligo sequence. The single strand circle DNA (ssCir DNA) was formatted as the final library. The final library was quantitatedin two ways in order to ensure the high quality of the sequencing data: Determined the average molecule length used the Agilent 2100 bioanalyzer instrument, quantified library used real-time quantitative PCR (qPCR). The final library was amplified with phi29 (Thermo Fisher Scientific,MA, USA) to make DNA nanoball (DNB) which had more than 300 copies of one molecular, DNBs were loaded into the patterned nanoarray and single end 50 bases reads were generated on BGISEQ500 platform (BGI-Shenzhen, China).

**1.11** | **Transcriptomic analysis**

SOAPnuke (v1.5.2)(R. Li et al., 2008) was used to filter the sequencing data: 1) Removing reads containing sequencing adapter, 2) Removing reads whose low-quality base ratio (base quality less than or equal to 5) is more than 20%, 3) Removing reads whose unknown base ('N' base) ratio is more than 5%, afterwards clean reads were obtained and stored in FASTQ format. The clean reads were mapped to the reference genome using HISAT2 (v2.0.4) (Kim et al., 2015). Bowtie2 (v2.2.5) (Langmead et al., 2012) was applied to align the clean reads to the reference coding gene set, then expression level of gene was calculated by RSEM (v1.2.12) (B. Li et al., 2011). The differentially expressed gene (DEG) analysis (adjusted *p* < 0.05) was implemented by DESeq2(v1.4.5) (Love et al., 2014). Metascape (Zhou et al., 2019) (http://metascape.org) was used to analysis DEGs in mCherry group and hTau N368 group mice. Gene ontology (GO) and Kyoto encyclopedia of genes and genomes (KEGG) enrichment analysis of DEGs were analyzed using Dr. Tom (BGI, Shenzhen, China) with a corrected *p* value < 0.05 is considered significant. Gene network analysis were performed using STRING database (https://string-db.org/). The Molecular Complex Detection （MCODE） algorithm were applied to identify densely connected network components(Bader et al., 2003).

**1.12** | **Behavioral test**

The open field test (OFT) was performed in a 40 × 40 × 40 cm (length × width × height) chamber. Mice were gently placed in the center of the chamber and allowed to explore freely, then recorded by a video camera for 10 min. The total distance traveled and the amount time spent in the center area of the chamber were calculated in 10 min time bins with ANYMAZE software (O’Hara & Co., Japan).

The Elevated plus maze (EPM) consisted of two closed arms (25 × 5 × 15 cm (H)), two open arms (25 × 5 × 0.5 cm (H)) and a central platform (5 × 5 cm). The maze was elevated 50 cm above the floor. Each mouse was individually placed in the center area at the start of a trial and allowed to explore freely in the maze for 5 min. The duration in open arms, the entries to the open arms, and the total distance traveled were recorded by the video tracking system (Chengdu Taimeng Software Co. Lid, China).

The object recognition test (ORT) is based on rodents’ spontaneous preference for novelty and ability to remember previously encountered objects (Leger et al., 2013). Prior to training, mice were placed in an open-field arena (40 x 40 x 40 cm) for habituation and allowed to explore the arena for 10 min. The next day, two identical objects were placed in the middle of the open-field arena, and each mouse allowed to explore them for 10 min. The time exploring the two objects was scored. During testing, one object was replaced with a novel object, but the location remained the same as during training. Recognition memory was tested 24 h after the training phase, mice were allowed to explore for 10 min, and the time exploring each object was recorded. The open field was cleaned with 75% ethanol between the introductions of each mouse. Videos were recorded and analyzed using ANYMAZE software (O’Hara & Co., Japan). The discrimination index toward novel object was calculated as the (D.I. = (T_novel_ – T_familiar_) / (T_novel_ + T_familiar_) x 100%).

The object location test (OLT) is based on the spontaneous tendency of rodents, previously exposed to two identical objects, later to explore the object that has been placed in a new location, rather than the non-displaced object (Murai et al., 2007). The procedure, equipment and analyses were similar to those described for the object recognition test, but the pattern inside the open field was removed. One day after habituation, two identical objects were placed in the open field, and mice were allowed to explore them for 10 min. The time exploring the two objects was scored. Spatial memory was tested 24 h later when one of the objects was moved to a novel position. Mice were allowed to explored for 10 min. The time exploring the displaced object was calculated as the discrimination index (D.I. = (T_displaced_ – T_unmoved_) / (T_displaced_ + T_unmoved_) x 100%)

The contextual fear conditioning (CFC) test was performed as previously described (Liu et al., 2017). For training, each mouse was placed in a chamber measuring 23 x 23 x 30 cm, with a metal wire floor and transparent plastic wall, set in a white soundproof cubicle. After 3 min, mice received three mild foot shock (0.65 mA for 2 s, 60 s rest for a trial). Chamber was cleaned with 75% alcohol to eliminate any residual odor. To assess contextual memory, the mice were placed back in the training context 24 h post-training for 3 min without an electric foot shock. The activity and freezing behavior of the animals, which was defined as a complete absence of movement, were recorded and analyzed by ANYMAZE software (O’Hara & Co., Japan).

The contextual discrimination training was performed as previously described (Zheng et al., 2020). Behavioral training began 5 days after the CFC, using a conditioning chamber (23 x 23 x 30 cm). The chamber consisted of the floor made of 32 stainless steel rods connected to a shock generator and a fan producing white noise (60dB). On day 1-3, each mouse was placed in the chamber (context A, paired with an additional white noise delivered through a small loudspeaker), and allowed to explore for 3 min. Then a single footshock (0.65 mA, 2 s) was delivered. Mice were allowed to stay in the chamber for another 1 min. The chamber was cleaned between each animal with 75% ethanol. On the subsequent day (day 4), mice were first placed in a very similar context (context B, floor of steel rods, no white noise, blue lighting, a plastic A-frame insert, and *Lanyueliang* cleaning the box between animals) from context A, allowed to freely explore for 3 min, and subsequently placed in the context A about 5 h later, allowed to freely explore for another 3 min. In the following days, mice were trained to discriminate context B from context A (Nakashiba et al., 2012). In context A, mice received a single footshock (0.65 mA, 2 s) after 3 min free-exploring, and allowed to stay in the chamber for another 1 min after the footshock. In context B, mice were placed in the chamber for 4 min, but no footshock was delivered after the first 3 min free-exploring. The time of freezing behavior was measured for the first 3 min every day. The training procedure followed a double alternation schedule, i.e., B-A, A-B, A-B, etc. Freezing behavior was recorded and analyzed by ANYMAZE software (O’Hara & Co., Japan).

**1.13** | **CNO delivery**

Clozapine-N-oxide (CNO, MedChemExpress, Catalog number 34233-69-7) was dissolved in dimethylsulfoxide (DMSO) at 1% final concentration, and then diluted with PBS to 1 mg/ml for intraperitoneal (i.p.) injections. For i.p. injections, 1 mg/kg of CNO was delivered, followed by behavior 30 min after the injection.

**1.14** | **Statistical analysis**

Data were presented as means ± SEM unless otherwise stated. All data were analyzed and plotted using GraphPad Prism (GraphPad Software), SPSS Statistics (IBM) or MATLAB (MathWorks). Unpaired or paired two-tailed t tests, ANOVA (one-way, two-way, or repeated-measures), two-tailed Fisher’s exact tests, Mann-Whitney tests, and post hoc Tukey’s multiple comparisons tests were used (as illustrated in figure legends), with **p* < 0.05 considered as statistically significant.

**References**

Bader, G. D., & Hogue, C. W. (2003). An automated method for finding molecular complexes in large protein interaction networks. *BMC Bioinformatics, 4*, 2. <http://doi.org/10.1186/1471-2105-4-2>

Bragin, A., Jando, G., Nadasdy, Z., van Landeghem, M., & Buzsaki, G. (1995). Dentate EEG spikes and associated interneuronal population bursts in the hippocampal hilar region of the rat. *J Neurophysiol, 73*(4), 1691-1705. <http://doi.org/10.1152/jn.1995.73.4.1691>

Du, H., Deng, W., Aimone, J. B., Ge, M., Parylak, S., Walch, K., . . . Mu, Y. (2016). Dopaminergic inputs in the dentate gyrus direct the choice of memory encoding. *Proc Natl Acad Sci U S A, 113*(37), E5501-5510. <http://doi.org/10.1073/pnas.1606951113>

Ge, M., Song, H., Li, H., Li, R., Tao, X., Zhan, X., . . . Mu, Y. (2019). Memory Susceptibility to Retroactive Interference Is Developmentally Regulated by NMDA Receptors. *Cell Rep, 26*(8), 2052-2063 e2054. <http://doi.org/10.1016/j.celrep.2019.01.098>

Hagihara, H., Toyama, K., Yamasaki, N., & Miyakawa, T. (2009). Dissection of hippocampal dentate gyrus from adult mouse. *J Vis Exp*(33). <http://doi.org/10.3791/1543>

Kim, D., Langmead, B., & Salzberg, S. L. (2015). HISAT: a fast spliced aligner with low memory requirements. *Nat Methods, 12*(4), 357-360. <http://doi.org/10.1038/nmeth.3317>

Langmead, B., & Salzberg, S. L. (2012). Fast gapped-read alignment with Bowtie 2. *Nat Methods, 9*(4), 357-359. <http://doi.org/10.1038/nmeth.1923>

Leger, M., Quiedeville, A., Bouet, V., Haelewyn, B., Boulouard, M., Schumann-Bard, P., & Freret, T. (2013). Object recognition test in mice. *Nat Protoc, 8*(12), 2531-2537. <http://doi.org/10.1038/nprot.2013.155>

Li, B., & Dewey, C. N. (2011). RSEM: accurate transcript quantification from RNA-Seq data with or without a reference genome. *BMC Bioinformatics, 12*, 323. <http://doi.org/10.1186/1471-2105-12-323>

Li, R., Li, Y., Kristiansen, K., & Wang, J. (2008). SOAP: short oligonucleotide alignment program. *Bioinformatics, 24*(5), 713-714. <http://doi.org/10.1093/bioinformatics/btn025>

Liu, E., Xie, A. J., Zhou, Q., Li, M., Zhang, S., Li, S., . . . Wang, J. Z. (2017). GSK-3beta deletion in dentate gyrus excitatory neuron impairs synaptic plasticity and memory. *Sci Rep, 7*(1), 5781. <http://doi.org/10.1038/s41598-017-06173-4>

Love, M. I., Huber, W., & Anders, S. (2014). Moderated estimation of fold change and dispersion for RNA-seq data with DESeq2. *Genome Biol, 15*(12), 550. <http://doi.org/10.1186/s13059-014-0550-8>

Murai, T., Okuda, S., Tanaka, T., & Ohta, H. (2007). Characteristics of object location memory in mice: Behavioral and pharmacological studies. *Physiol Behav, 90*(1), 116-124. <http://doi.org/10.1016/j.physbeh.2006.09.013>

Nakashiba, T., Cushman, J. D., Pelkey, K. A., Renaudineau, S., Buhl, D. L., McHugh, T. J., . . . Tonegawa, S. (2012). Young dentate granule cells mediate pattern separation, whereas old granule cells facilitate pattern completion. *Cell, 149*(1), 188-201. <http://doi.org/10.1016/j.cell.2012.01.046>

Schindelin, J., Arganda-Carreras, I., Frise, E., Kaynig, V., Longair, M., Pietzsch, T., . . . Cardona, A. (2012). Fiji: an open-source platform for biological-image analysis. *Nat Methods, 9*(7), 676-682. <http://doi.org/10.1038/nmeth.2019>

Zheng, J., Jiang, Y. Y., Xu, L. C., Ma, L. Y., Liu, F. Y., Cui, S., . . . Yi, M. (2017). Adult Hippocampal Neurogenesis along the Dorsoventral Axis Contributes Differentially to Environmental Enrichment Combined with Voluntary Exercise in Alleviating Chronic Inflammatory Pain in Mice. *J Neurosci, 37*(15), 4145-4157. <http://doi.org/10.1523/JNEUROSCI.3333-16.2017>

Zheng, J., Li, H. L., Tian, N., Liu, F., Wang, L., Yin, Y., . . . Wang, J. Z. (2020). Interneuron Accumulation of Phosphorylated tau Impairs Adult Hippocampal Neurogenesis by Suppressing GABAergic Transmission. *Cell Stem Cell, 26*(3), 331-345 e336. <http://doi.org/10.1016/j.stem.2019.12.015>

Zhou, Y., Zhou, B., Pache, L., Chang, M., Khodabakhshi, A. H., Tanaseichuk, O., . . . Chanda, S. K. (2019). Metascape provides a biologist-oriented resource for the analysis of systems-level datasets. *Nat Commun, 10*(1), 1523. <http://doi.org/10.1038/s41467-019-09234-6>
