# Supplementary material for: Alzheimer‐like tau accumulation in dentate gyrus mossy cells induces spatial cognitive deficits by disrupting multiple memory‐related signaling and inhibiting local neural circuit
Source: Aging Cell. 2022 Mar 31;21(5):e13600. doi: 10.1111/acel.13600 (PMC9124302; doi:10.1111/acel.13600)
Supplement: Supplementary file 2 — Figure S1‐S8 [file ACEL-21-e13600-s001.docx]

**Supplemental Figures**

**Figure S1**


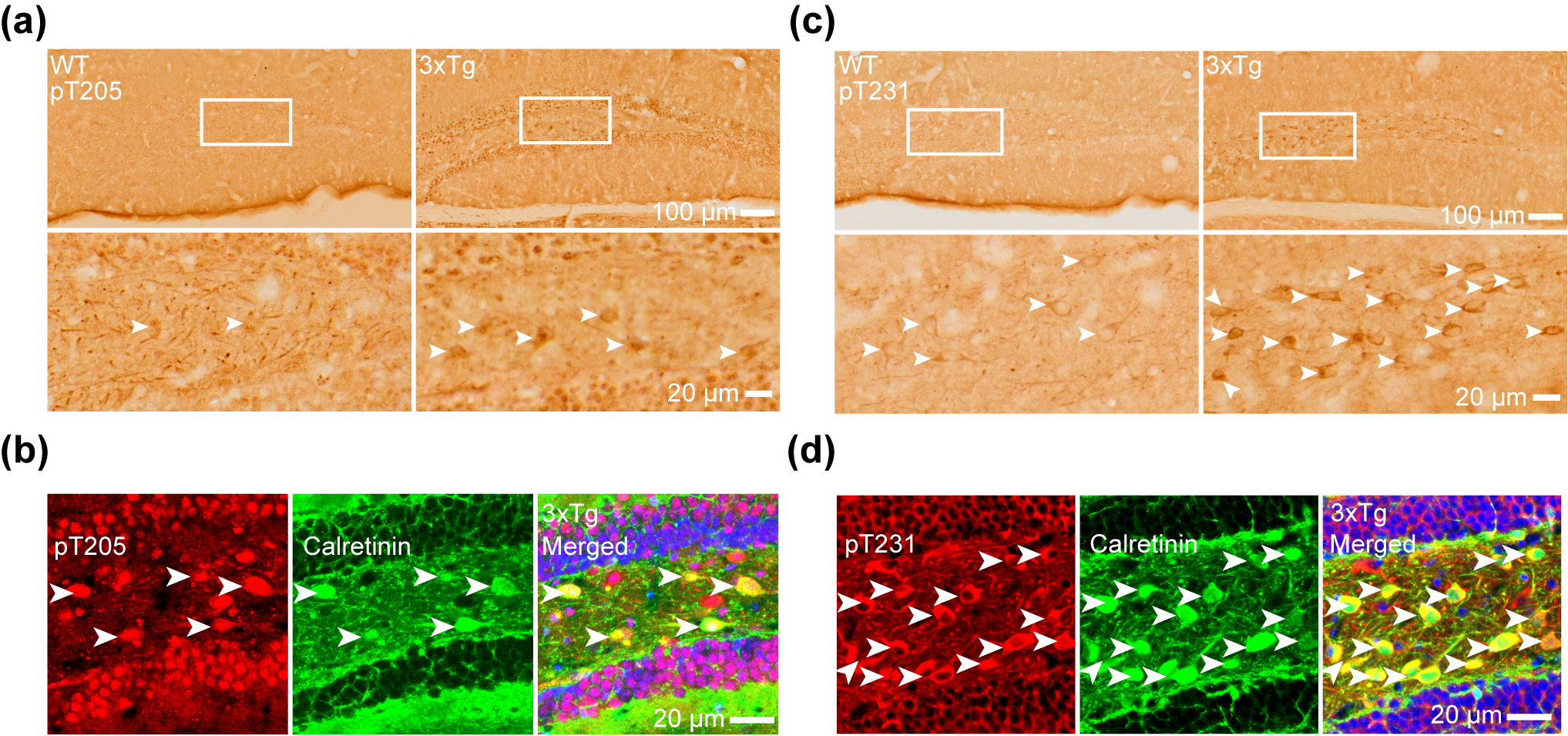


**FIGURE S1.** Prominent accumulation of hyperphosphorylated tau (pTau) in the hilus mossy cells of hippocampal dentate gyrus in 3xTg-AD mice. (a, c) Representative images showing prominent accumulation of phosphor-Thr205 (pT205, arrows in panel a) and phosphor-Thr231 (pT231, arrows in panel c) in the hilus of hippocampal dentate gyrus of 7-month 3xTg-AD mice. Images were scanned and automatically spliced using the VS-ASW-S6 software (Olympus). Scale bars were as indicated in each image. (b, d) Most pTau-positive cells in the hilus were identified as mossy cells, as indicated by co-labeling of pT205 (arrows in panel b) or pT231 (arrows in panel d) with calretinin (mossy cell marker). Scale bars, 20 μm

**Figure S2**


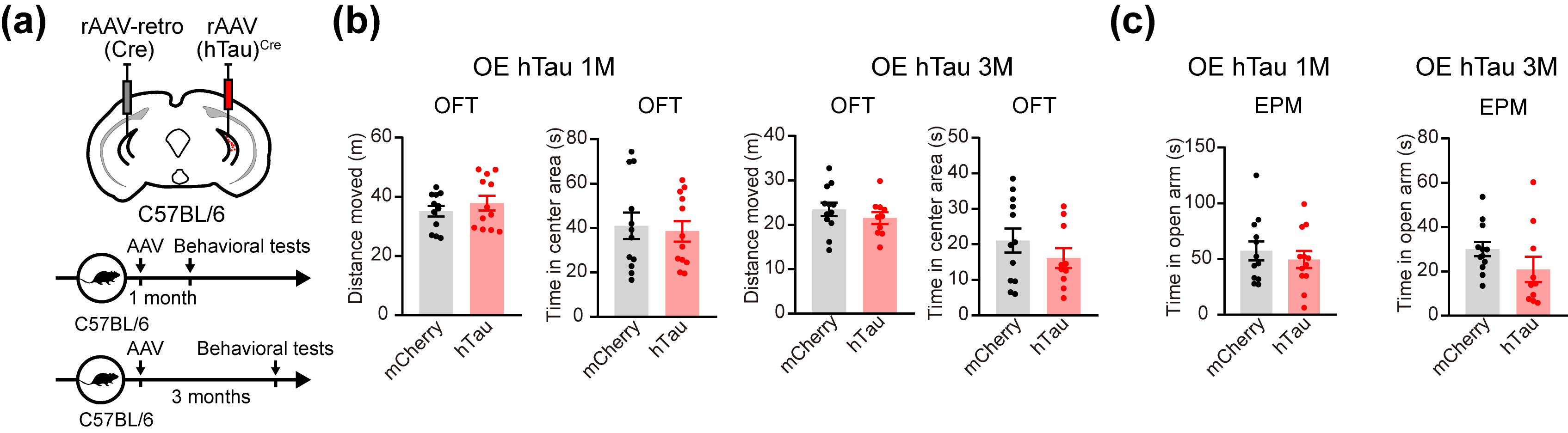


**FIGURE S2.** Related to Figure 1. Unilateral mossy cell-specific overexpressing wild-type full length hTau in C57BL/6 mice does not induce anxiety-like phenotype. (a) Strategies used for mossy cell-specific overexpression hTau in C57BL/6 mice was achieved as described in Figure 1a. (b, c) Mossy cell overexpression hTau for one month or three months in C57BL/6 mice did not induce anxiety-like phenotype measured by open field test (OFT, b) and elevated plus maze (EPM, c), respectively. Unpaired t tests, **p* < 0.05. n = 10~12 mice in each group. Data were represented as mean ± SEM

**Figure S3**


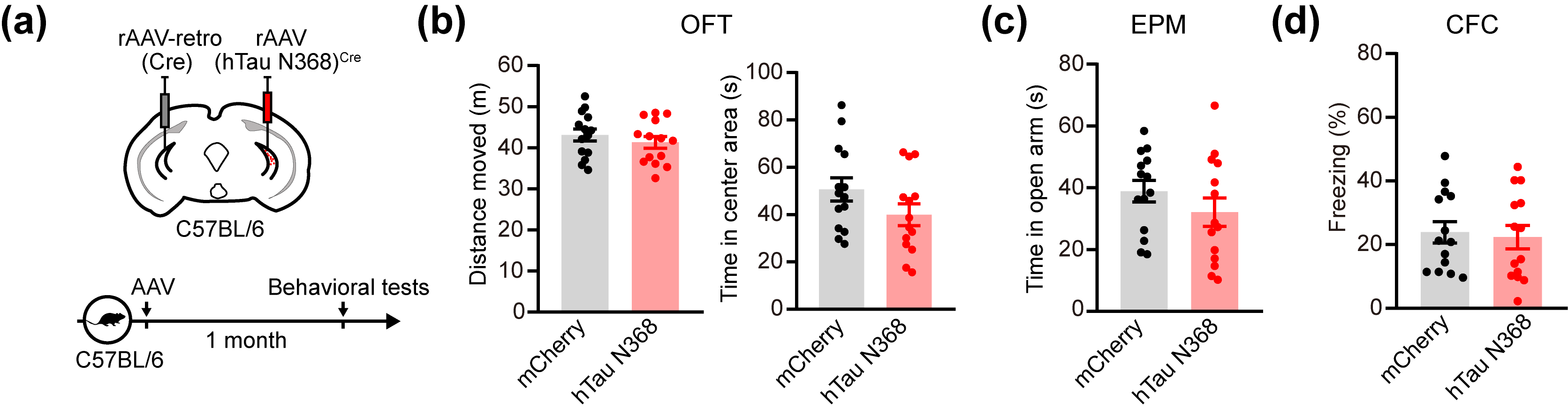


**FIGURE S3.** Related to Figure 3. Unilateral mossy cell-specific overexpressing hTau N368 in C57BL/6 mice does not induce anxiety-like phenotype and contextual fear memory deficit. (a) Mossy cell-specific overexpressing hTau N368 in C57BL/6 mice was achieved as described in Figure 2a, and behavioral tests were performed after mossy cell overexpressing hTau N368 for one month. (b, c) Unilateral mossy cell-specific overexpressing hTau N368 for one month did not induce anxiety-like behavior evidenced by unchanged distance moved and time in center area during OFT (b) and time in open arm in EPM (c) tests, respectively. (d) Unilateral mossy cell-specific overexpressing hTau N368 for one month did not induce contextual fear memory deficit measured by contextual fear conditioning (CFC). Unpaired t tests, **p* < 0.05. n = 14 mice in each group. Data were represented as mean ± SEM

**Figure S4**


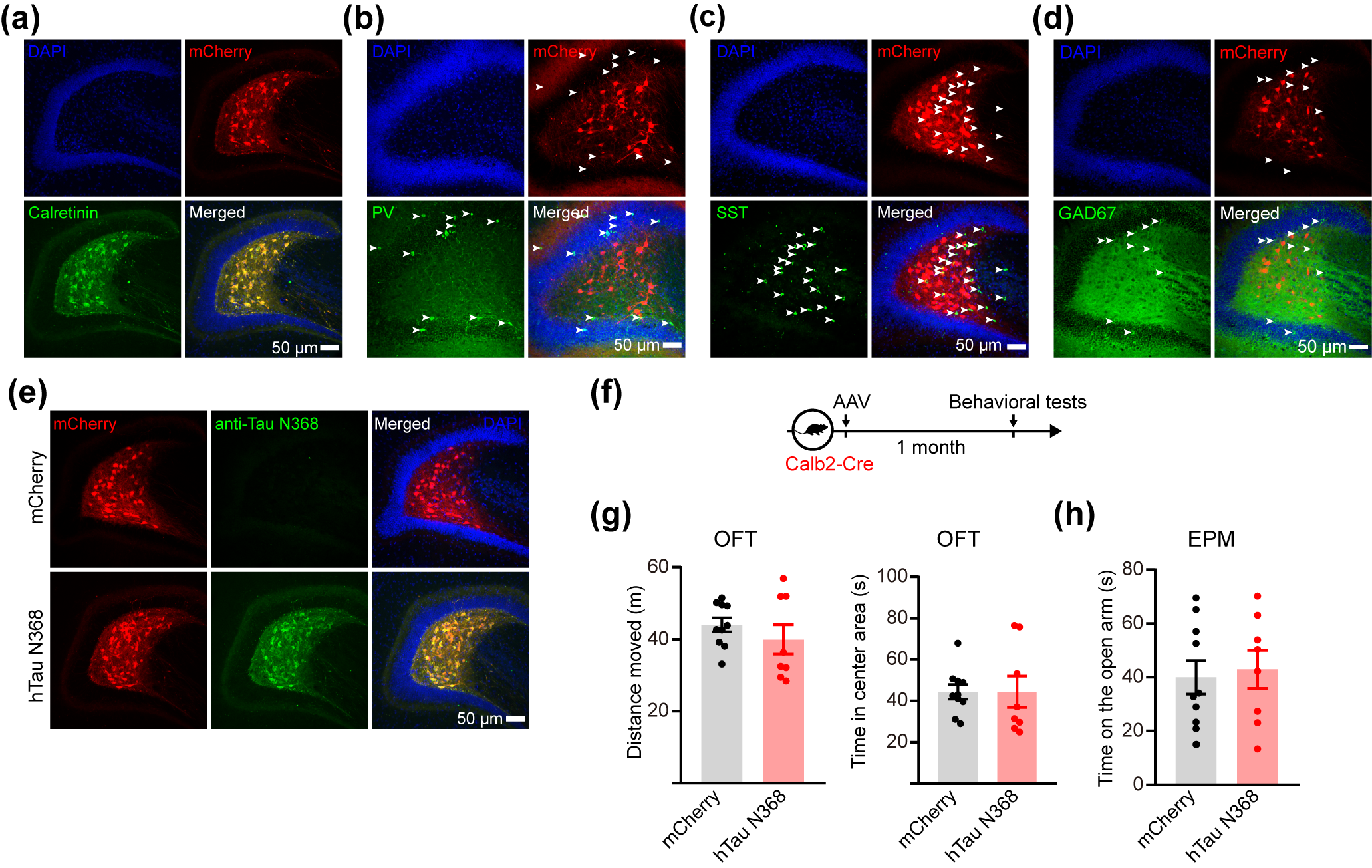


**FIGURE S4.** Related to Figure 3. Bilateral mossy cell-specific overexpressing hTau N368 in Calb2-Cre mice does not induce anxiety-like phenotype. (a) The mCherry expression by AAV-DIO-hTau N368-mCherry were co-labeled with calretinin (mossy cell marker). Scale bar, 50 μm. (b-d) Mossy cell overexpression hTau N368 in Calb2-Cre, the mCherry positive cells were not co-labeled with PV (arrows in panel b), SST (arrows in panel c) and GAD67 (arrows in panel d). Scale bars, 50 μm. (e) Mossy cell overexpression hTau N368 in Calb2-Cre, confirmed by co-labeled with anti-Tau N368. Scale bar, 50 μm. (f-h) Mossy cell overexpression hTau N368 in Calb2-Cre mice showing no anxiety-associated behavioral measured by OFT (g) and EPM (h). Unpaired t tests, **p* < 0.05. n = 8~10 mice in each group. Data were represented as mean ± SEM

**Figure S5**


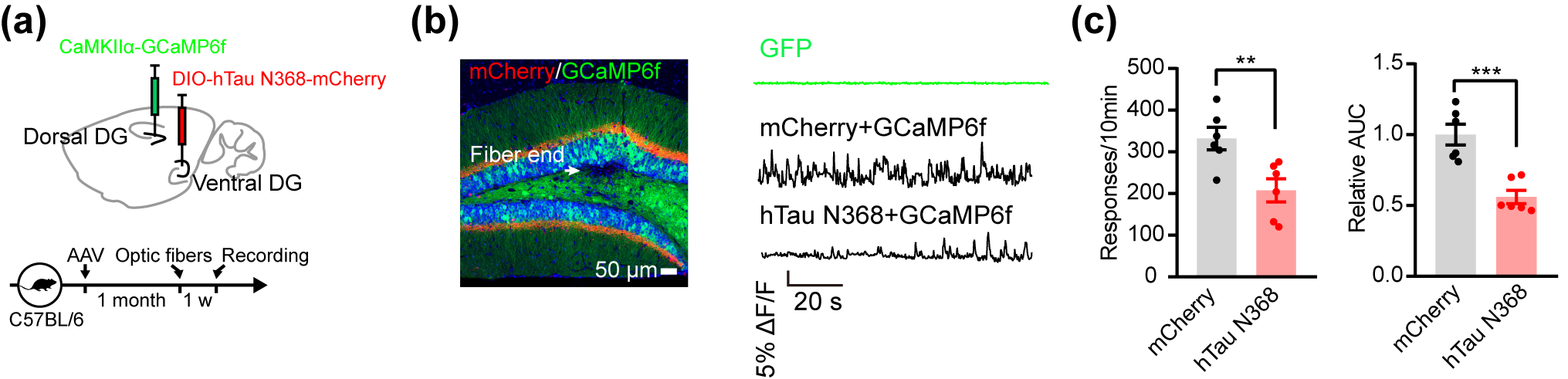


**FIGURE S5.** Related to Figure 4. Unilateral mossy cell-specific overexpressing hTau N368 in C57BL/6 mice decreased calcium response in DG excitatory neurons. The AAV-carried hTau N368 and GCaMP6f were respectively infused into ventral and dorsal DG subsets of C57BL/6 mice for one month, then the optic fibers were implanted and *in vivo* optic fiber recording was carried out after 1 week. The schematics (a), representative image (b) and ΔF/F signal presented by using 5% ΔF/F as threshold (c) were shown. Scale bar, 50 μm. Unpaired t tests, n = 6 mice in each group, **p* < 0.05, ***p* < 0.01, ****p* < 0.001. Data were represented as mean ± SEM

**Figure S6**


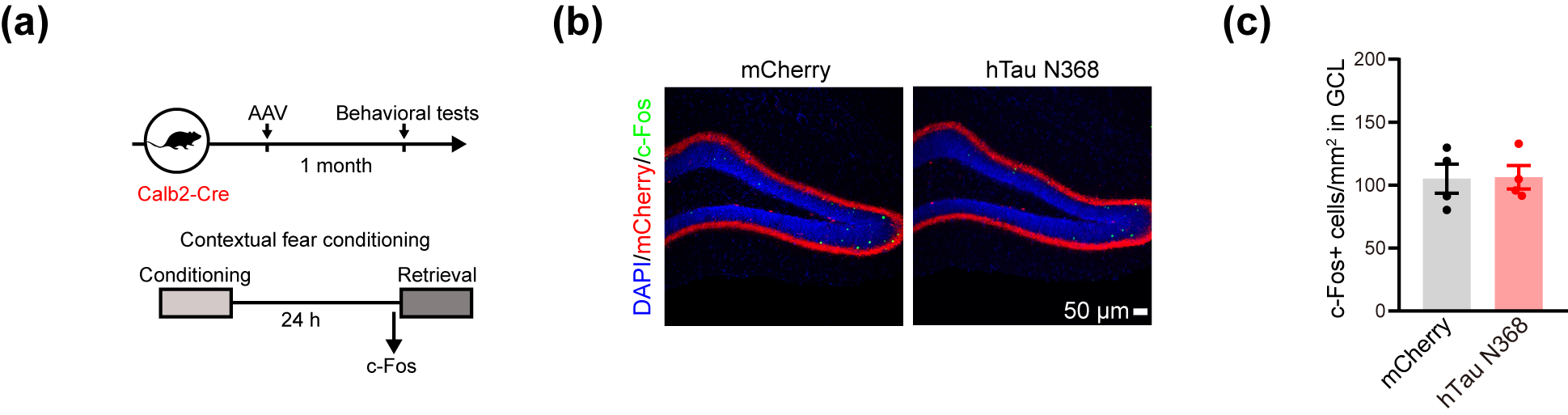


**FIGURE S6.** Related to Figure 4. Mossy cell-specific overexpressing hTau N368 in Calb2-Cre mice does not alter the basal level of c-Fos in dDG. Mossy cell-specific overexpression hTau N368 in Calb2-Cre mice for one month didn’t change the basal expression of c-Fos in granule cell layer (GCL) shown by the numbers of c-Fos+ granule cells measured at the time immediately before the retrieval test of contextual fear conditioning test. The schematics of the experimental procedure (a), representative co-immunofluorescence images (b) and the quantitative analysis (c). Unpaired t tests, n = 4 mice in each group, **p* < 0.05. Scale bar, 50 μm. Data were represented as mean ± SEM

**Figure S7**


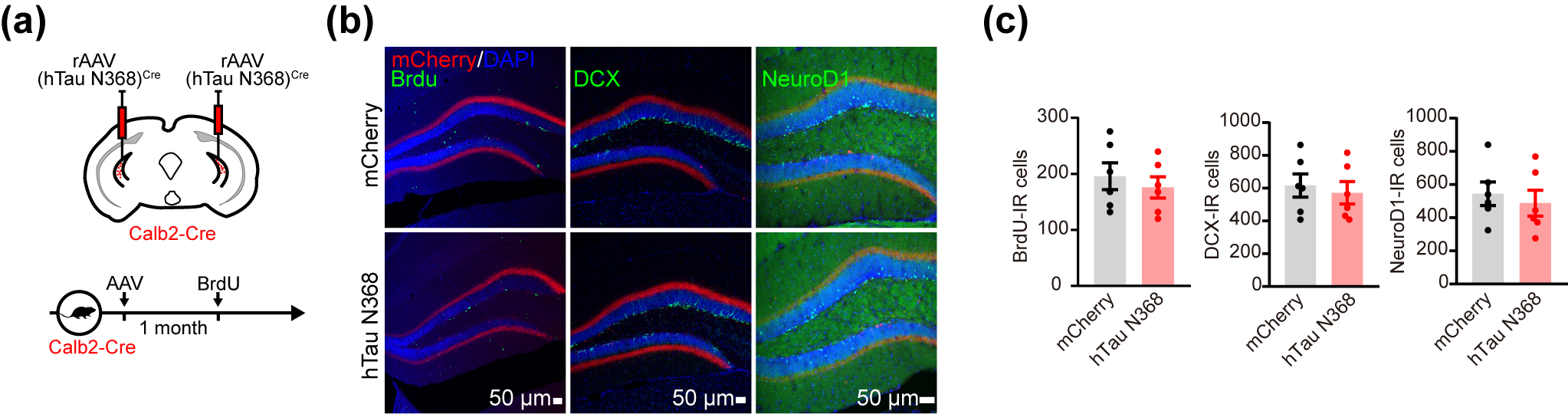


**FIGURE S7.** Mossy cell-specific overexpressing hTau N368 in Calb2-Cre mice does not affect adult hippocampal neurogenesis. The AAV-carried hTau N368 was injected into the ventral DG subsets of Calb2-Cre mice for one month, then BrdU (0.2 ml，10 mg/ml) was injected intraperitoneally 5 days before sacrifice of the mice (a). The number of BrdU-, DCX-, and NeuroD1-immunoreactive cells in dDG subset was measured by co-immunofluorescence staining (b) and quantitative analysis (c). Unpaired t tests, n = 6 mice in each group, **p* < 0.05. Scale bars, 50 μm. Data were represented as mean ± SEM

**Figure S8**


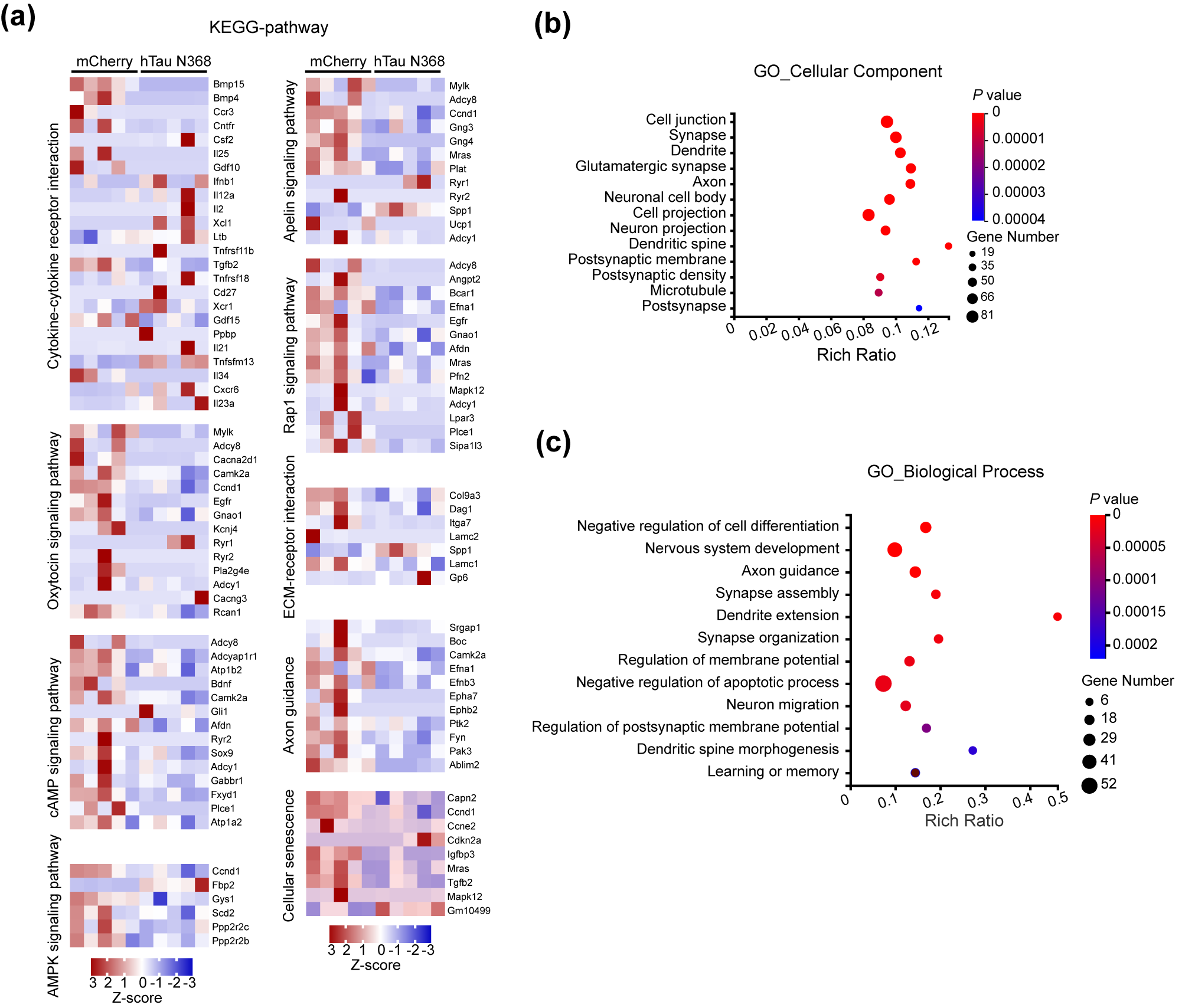


**FIGURE S8**. Related to Figure 5. Mossy cell-specific overexpressing hTau N368 alters gene expression involved in multiple pathways, cellular component and biological process. (a) Heatmap showing gene expression of DEGs in Cytokine-cytokine interaction, Oxytocin signaling pathway, Apelin signaling pathway, cAMP signaling pathway, Rap1 signaling pathway, ECM-recptor interaction, Axon guidance, AMPK signaling pathway and Cellular senescence. (b-c) Gene Ontology (GO) analysis Cellular Component (b) and Biological Process (c) enriched for DEGs between hTau N368 and mCherry group. n = 5 mice for each group
